# Immunomodulatory Activities of Emerging Rare Ginsenosides F1, Rg5, Rk1, Rh1, and Rg2: From Molecular Mechanisms to Therapeutic Applications

**DOI:** 10.3390/ph18101529

**Published:** 2025-10-11

**Authors:** Chang-Eui Hong, Su-Yun Lyu

**Affiliations:** 1 Department of Pharmacy, College of Pharmacy; Sunchon National University, Suncheon 57922, Republic of Korea; 2 Smart Beautytech Research Institute, Sunchon National University, Suncheon 57922, Republic of Korea; 3 Research Institute of Life and Pharmaceutical Sciences, Sunchon National University, Suncheon 57922, Republic of Korea

**Keywords:** rare ginsenosides, ginsenoside F1, ginsenoside Rg5, ginsenoside Rk1, ginsenoside Rh1, ginsenoside Rg2

## Abstract

Ginsenosides, the primary bioactive components of *Panax ginseng*, have demonstrated significant immunomodulatory potential. While major ginsenosides have been extensively studied, rare ginsenosides produced through deglycosylation, heating, and steaming show enhanced biological activities with improved bioavailability. This review aimed to comprehensively analyze the immunomodulatory mechanisms, structure-activity relationships (SARs), therapeutic applications, and clinical translation strategies of five emerging rare ginsenosides: F1, Rg5, Rk1, Rh1, and Rg2. We conducted a comprehensive literature review examining the production methods, immunological effects, molecular mechanisms, pharmacokinetics, safety profiles, and clinical applications of these five compounds. Analysis focused on chemical structures, immune cell modulation, signaling pathways, disease model efficacy, and bioavailability enhancement strategies. Ginsenoside F1 uniquely demonstrated immunostimulatory effects, enhancing natural killer (NK) cell cytotoxicity and macrophage phagocytosis through mitogen-activated protein kinase (MAPK)/nuclear factor-κB (NF-κB) activation. Conversely, Rg5, Rk1, Rh1, and Rg2 exhibited anti-inflammatory properties via distinct mechanisms: Rg5 through Toll-like receptor 4 (TLR4)/NF-κB inhibition, Rk1 via triple pathway modulation (NF-κB, p38 MAPK, signal transducer and activator of transcription (STAT)), Rh1 by selective p38 MAPK and STAT1 inhibition, and Rg2 through modulation of both central nervous system (neuroinflammation) and peripheral organ systems. Structure-activity analysis revealed that sugar moiety positions critically determine immunological outcomes. Crucially, advanced delivery systems including nanostructured lipid carriers, self-microemulsifying systems, and specialized liposomes have overcome the major translational barrier of poor bioavailability, achieving up to 2.6-fold improvements and enabling clinical development. Safety assessments demonstrated favorable tolerability profiles across preclinical and clinical studies. These five rare ginsenosides represent promising immunomodulatory agents with distinct therapeutic applications. F1’s unique immunostimulatory properties position it for cancer immunotherapy, while the complementary anti-inflammatory mechanisms of Rg5, Rk1, Rh1, and Rg2 offer opportunities for precision medicine in inflammatory diseases. Advanced formulation technologies and optimized production methods now enable their significant clinical translation potential, providing promising therapeutic options for immune-related disorders pending further development.

## 1. Introduction

Ginseng, particularly *Panax ginseng* C.A. Meyer (Korean ginseng), has been used as a valuable medicinal herb in traditional Asian medicine for thousands of years [1,2,3]. The herb has been traditionally recognized for its ability to restore vital energy and employed as an adaptogen to normalize body functions and strengthen systems compromised by stress [4,5]. This traditional use as an immune-regulating agent has been particularly prominent in Korea, China, and Japan, where ginseng preparations have been prescribed for preventing various diseases and enhancing the body’s defense mechanisms [6,7,8]. Modern pharmacological research has validated these traditional applications, identifying ginsenosides—a unique class of dammarane-type triterpene saponins—as the primary bioactive components responsible for ginseng’s therapeutic effects [3,9,10]. Ginsenosides demonstrate immunomodulatory activities through multiple mechanisms, including modulation of macrophage function, regulation of inflammatory cytokines such as tumor necrosis factor alpha (TNF-α) and interleukin (IL)-6, enhancement of natural killer cell activity, and regulation of nuclear factor-kappa B (NF-κB) and mitogen-activated protein kinase (MAPK) signaling pathways [11,12,13]. Approximately 200 distinct compounds have been isolated from *Panax ginseng* to date [3], with numerous ginsenosides identified and characterized, establishing it as one of the most chemically complex medicinal plants [9,14].

Ginsenosides are classified into protopanaxadiol (PPD), protopanaxatriol (PPT), C17 side-chain varied (C17SCV), and oleanolic acid (OA) types based on their aglycone structures [15]. Based on abundance of cultivated ginseng, these compounds are traditionally divided into major ginsenosides (Rb1, Rb2, Rc, Rd, Re, Rg1) that constitute the bulk of total ginsenoside content, and rare ginsenosides that exist in much lower quantities or are produced through biotransformation [16]. These rare ginsenosides—including F1, F2, Rg3, Rg5, Rk1, Rh1, Rh2, Rg2, and Compound K—exhibit superior pharmacological activity and bioavailability compared to major ginsenosides due to their reduced molecular weight and enhanced membrane permeability [16,17]. Major to rare ginsenoside conversion occurs through multiple pathways [18]: intestinal bacteria metabolize PPD-type ginsenosides through stepwise deglycosylation following the pathway Rb1/Rb2/Rc → Rd → F2 → Compound K [19,20], while heat processing during red ginseng production generates Rg3, Rg5, and Rk1 through hydrolysis, dehydration, and isomerization [21,22]. Among these rare ginsenosides, F1, Rg5, Rk1, Rh1, and Rg2 have emerged as particularly promising immunomodulators with distinct mechanisms of action [17,23].

Among rare ginsenosides, recent research has particularly highlighted those with unique immunomodulatory mechanisms. Ginsenoside F1 distinguishes itself through immunostimulatory properties, enhancing natural killer (NK) cell cytotoxicity via insulin-like growth factor-1 (IGF-1) receptor-dependent mechanism [24] and promoting angiogenesis for cerebral ischemia. The compound crosses the blood–brain barrier within 2 h, protecting against amyloid beta-induced toxicity through insulin-degrading enzyme (IDE) and neprilysin (NEP) regulation [25,26]. Its therapeutic efficacy extends to eosinophilic inflammation, where it attenuates chronic rhinosinusitis by promoting NK cell function, contrasting with dexamethasone’s macrophage suppression [27].

In contrast to F1’s immunostimulatory effects, the other four rare ginsenosides primarily exhibit anti-inflammatory and cytoprotective properties. Ginsenoside Rg5 acts as a direct toll-like receptor 4 (TLR4) antagonist and demonstrates potent anti-inflammatory activity, achieving particularly high tissue exposure in lung [28]. Ginsenoside Rk1 modulates multiple inflammatory pathways simultaneously, providing protection against cisplatin-induced nephrotoxicity [22]. Ginsenoside Rh1 exhibits anti-inflammatory and antioxidant effects by activating the nuclear factor erythroid 2-related factor-2 (Nrf2)/heme oxygenase (HO)-1 signaling pathway, protecting vascular endothelial cells [29]. Rg2 demonstrates multiple protective effects including cardioprotection against drug-induced toxicity, inhibition of osteoclastogenesis, and antiarrhythmic effects without oral toxicity [30,31,32]. These complementary mechanisms suggest potential for both individual therapeutic applications and synergistic combinations.

While *Panax ginseng* contains diverse bioactive compounds, critical evaluation reveals significant therapeutic limitations in non-ginsenoside components. Ginseng polysaccharides exhibit potent immunostimulation including B cell mitogenic activity and NK cell activation through TLR2/TLR4 and MAPK/NF-κB pathways, but suffer from poor oral bioavailability due to high molecular weight, requiring parenteral administration for clinical efficacy [33,34]. Polyacetylenes such as panaxynol and panaxydol demonstrate cytotoxicity but lack selectivity between normal and malignant cells, limiting clinical application [2]. Phenolic compounds primarily contribute antioxidant rather than direct immunomodulatory effects [35]. In contrast, ginsenosides overcome these limitations through intestinal biotransformation by gut microbiota into absorbable metabolites with enhanced bioactivity [3]. Among ginsenosides, Rg3 and Compound K have achieved commercial development with clinical trials established, supported by numerous reviews documenting their anticancer and anti-inflammatory effects [23,36,37]. However, five rare ginsenosides—F1, Rg5, Rk1, Rh1, and Rg2—warrant focused analysis based on: (1) distinct immunomodulatory properties from major ginsenosides; (2) representation of both PPD and PPT structural types; (3) recent research interest yet lacking comprehensive review; and (4) potential for therapeutic development based on emerging preclinical data.

This review comprehensively analyzes the chemical structures, immunomodulatory mechanisms, therapeutic applications in cancer and inflammatory diseases, and clinical translation strategies for these five emerging rare ginsenosides. By integrating structure–activity relationship (SAR) analysis with synergistic potential assessment, we provide a novel perspective addressing the critical gap between preclinical discoveries and therapeutic applications.

## 2. Chemical Structures and Pharmacological Properties

### 2.1. Structure, Biosynthesis, and Production

#### 2.1.1. Classification and Basic Structures

The five rare ginsenosides examined in this review—F1, Rg5, Rk1, Rh1, and Rg2—are derived from the dammarane triterpene skeleton through structural modifications. Based on their aglycone moieties, they are classified into PPD-type (Rk1, Rg5) and PPT-type (F1, Rh1, Rg2) ginsenosides [3,15].

PPD-type ginsenosides feature sugar attachments at C-3 and C-20 positions with hydroxyl groups at 3-β, 12-β, and 20 pro-S positions [38]. PPT-type ginsenosides possess an additional hydroxyl group at C-6, enabling glycosylation at both C-6 and C-20 positions [39,40].

**Figure 1:**
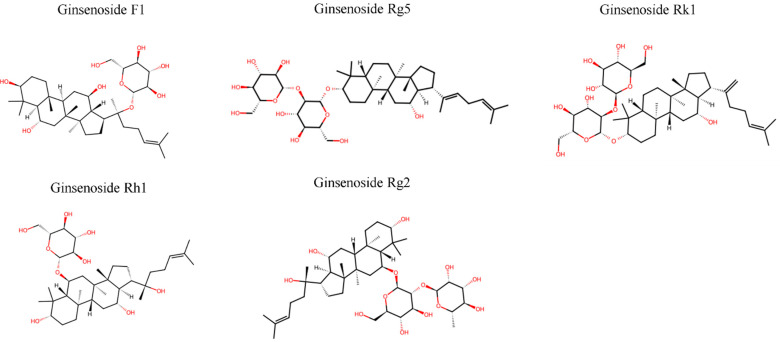
Chemical structures of five rare ginsenosides. PPD-type (Rk1, Rg5) and PPT-type (F1, Rh1, Rg2) compounds with different sugar moiety arrangements. Red portions indicate sugar moieties. Structures obtained from PubChem database. PPT, protopanaxatriol; PPD, protopanaxadiol.

Specifically, F1 contains a single glucose at C-20, Rg5 has glucose moieties at both C-3 and C-20 with dehydration at C-20(21), Rk1 possesses glucose at C-3 with dehydration at C-20(22), Rh1 features glucose at C-6, and Rg2 contains glucose at C-3 and rhamnose at C-6 (Figure 1). This structural distinction creates a fundamental paradox. While both PPD and PPT types generally exhibit anti-inflammatory properties through NF-κB and MAPK pathway inhibition [41,42], F1 uniquely stimulates immune responses via NK cell activation [25]. This phenomenon is directly linked to F1’s specific sugar positioning at C-20 without C-6 glycosylation.

#### 2.1.2. Formation Pathways and Production Technologies

The biosynthesis and metabolic origins of these rare ginsenosides vary considerably. F1 (molecular formula C_36_H_62_O_9_, molecular weight (MW) 638.87) is primarily formed through intestinal bacterial transformation of major ginsenosides Re and Rg1 via stepwise deglycosylation. The pathway proceeds as Re/Rg1 → Rg2 → Rh1 → F1, with α-L-rhamnosidase removing rhamnose from Rg2, followed by β-glucosidase removing the C-6 glucose from Rh1 [10,38,43]. This biotransformation can be efficiently achieved using commercial enzymes such as Cellulase KN, enabling production of 13.0 g F1 from 50 g substrate with 91.5% purity [44]. The enzymatic conversion can also be accomplished using β-glucosidase from *Lactobacillus brevis*, which exhibits ginsenoside-transforming capabilities through ketonization at the C-3 position [45].

In contrast, Rg5 (C_42_H_72_O_13_, MW 785.02) and Rk1 (C_42_H_72_O_13_, MW 785.02) are generated during the heat processing of ginseng through dehydration reactions at the C-20 position of Rg3, typically occurring during steaming at temperatures above 100 °C for 2–3 h [46,47]. These positional isomers differ in double bond location: Rg5 at C-20(21) and Rk1 at C-20(22), resulting from different hydroxyl elimination positions during thermal processing [48]. The formation of these dehydrated ginsenosides follows a specific pathway: major ginsenosides (Rb1, Rb2, Rc) → Rd → Rg3 → Rk1/Rg5, with the conversion rate and product distribution dependent on processing conditions such as temperature, time, and pH [47,49].

Rh1 (C_36_H_62_O_9_, MW 638.87) is produced through the removal of the terminal α-L-rhamnose at C-6 of Rg2 by α-L-rhamnosidase [38,50]. Rg2 (C_42_H_72_O_13_, MW 785.03), another PPT-type rare ginsenoside, is formed from major ginsenoside Re through partial deglycosylation [38]. The production of these compounds can be achieved through enzymatic transformation using special ginsenosidase type-I from *Aspergillus niger* g.848 [51], or through recombinant β-glucosidase expression in generally recognized as safe (GRAS) hosts [52].

#### 2.1.3. Production Technologies

Recent advances in biotransformation technologies have enabled more efficient production of these rare ginsenosides. Computer-guided enzyme engineering achieved a 13.88-fold increase in catalytic efficiency for F1 production through site-directed mutagenesis of β-glucosidase [53]. Heat processing converts Rg3 to Rg5 and Rk1 through selective dehydration [46,47]. Commercial enzyme preparations like Cellulase KN enable large-scale F1 production with over 90% purity [44]. These biotechnological advances not only improve yields but also allow for controlled production of specific structural variants with distinct biological activities.

From an industrial pharmaceutical perspective, the scalability and cost-effectiveness of these production methods vary significantly. Enzymatic conversion using immobilized β-glucosidases demonstrates superior selectivity and yields, achieving 80–100% conversion rates under mild conditions (pH 5–6, 30–50 °C) with minimal byproduct formation [54,55]. Recent advances in metal–organic framework (MOF)-based enzyme immobilization have enhanced economic viability, with systems maintaining 70–80% activity after 10 cycles of reuse [56,57]. While enzymatic methods require higher initial investment for enzyme production and purification, the ability to reuse immobilized enzymes and achieve complete substrate conversion makes them increasingly cost-effective for pharmaceutical-grade production [58]. In contrast, heat processing at 120 °C for 3–12 h offers simpler implementation with lower initial costs but suffers from reduced selectivity, higher energy consumption, and generation of unwanted byproducts [50,59]. For large-scale production, bioreactor systems employing engineered microorganisms such as Saccharomyces cerevisiae have achieved ginsenoside titers of 1.95–3.6 g/L through fed-batch fermentation, demonstrating industrial feasibility [60]. The choice between enzymatic and thermal processing ultimately depends on the target ginsenoside specificity, required purity, and production scale, with enzymatic methods increasingly preferred for pharmaceutical applications requiring high purity and specific rare ginsenoside profiles.

#### 2.1.4. Structure–Function Relationships

The sugar moiety positions and compositions play crucial roles in determining the immunomodulatory activities of these compounds. Most notably, the C-20 glucose position in F1 has been identified as essential for its unique NK cell activation properties, while the C-6 position is dispensable [25]. This SAR finding provides critical insight into why F1 alone among the rare ginsenosides enhances rather than suppresses immune responses. In contrast, the dehydration at C-20 positions in Rg5 and Rk1, creating double bonds at C-20(21) and C-20(22, respectively, correlates with their potent anti-inflammatory activities through direct interaction with molecular targets such as TLR4 [61].

The structural diversity among these five rare ginsenosides extends beyond simple glycosylation patterns. The stereochemistry at C-20 also plays a significant role, with both 20(S) and 20(R) epimers existing for compounds like Rg2, Rg3, and Rh1, each potentially exhibiting different biological activities [48,62]. Understanding these SARs are essential for rational drug design and the development of synthetic analogues with enhanced therapeutic potential.

The structural characteristics and immunomodulatory properties of these five rare ginsenosides are summarized in Table 1, highlighting their distinct molecular features and biological activities that form the basis for their therapeutic applications.

### 2.2. SAR for Immunomodulation

Building on the general structural features described above, this section examines the specific molecular mechanisms by which structural variations determine immunomodulatory outcomes.

#### 2.2.1. Sugar Position and Immune Activity

The position of sugar attachments plays a pivotal role in determining whether a ginsenoside will enhance or suppress immune responses. Ginsenoside F1 has been identified as a potent enhancer of NK cell cytotoxicity through an IGF-1-dependent mechanism, demonstrating the most potent activity among 15 different ginsenosides tested [25]. Specifically, the glucose at C-20 is essential for this immunostimulatory effect, while removal of the C-6 sugar maintains the activity, contrasting sharply with other ginsenosides where structural modifications typically lead to immunosuppression [25].

The biological activities of ginsenosides depend on both the diversity of sugar components and the number and location of sugar moieties [68]. These sugar positions influence not only solubility but also direct interactions with cellular receptors, determining whether a compound will stimulate or suppress immune responses [64].

#### 2.2.2. Anti-Inflammatory Mechanisms and Sugar Moiety Effects

The anti-inflammatory activities of rare ginsenosides demonstrate clear structure-dependent patterns. Ginsenoside Rg5 exhibits potent anti-inflammatory effects through direct inhibition of TLR4 signaling, suppressing lipopolysaccharide (LPS)-induced inflammatory responses in BV2 microglial cells by inhibiting the phosphorylation of phosphatidylinositol 3-kinase/protein kinase B (PI3K/AKT) and MAPKs, and reducing the DNA binding activities of NF-κB and activator protein-1 (AP-1) [64].

Ginsenosides Rk1 and Rg5 suppress high mobility group box 1 (HMGB1)-mediated septic responses through sirtuin 1 (SIRT1)-mediated deacetylation of HMGB1, inhibiting TNF-α and IL-6 production as well as NF-κB and extracellular signal-regulated kinase (ERK) 1/2 activation [66]. The dehydration at C-20 positions in these compounds appears to enhance their ability to interact with membrane receptors and modulate inflammatory pathways [69].

Additionally, the combination of ginsenosides Rg2 and Rh1 synergistically attenuates LPS-induced inflammation through selective inhibition of p38 MAPK and signal transducer and activator of transcription (STAT) 1 activation, while blocking protein kinase C delta (PKCδ) translocation to the plasma membrane [42].

#### 2.2.3. Stereochemistry and Biological Activity

The stereochemistry at C-20 significantly influences the biological activities of ginsenosides. The hydroxyl group at C-20 is spatially closer to the hydroxyl group at C-12 in the 20(S)-ginsenosides than their 20(R) counterparts, affecting their pharmacological properties [70]. This stereochemical difference results in distinct interactions with lipid membranes and cellular targets.

Pharmacokinetic studies have shown different behaviors between 20(R) and 20(S) epimers of ginsenosides including Rh1 [71], with stereospecificity extending to immunomodulatory effects where different epimers can induce autophagy and apoptosis in a stereoisomer-specific manner [41]. This suggests that the spatial orientation of functional groups is crucial for target recognition and binding.

It should be noted that many studies cited in this review do not specify whether 20(S) or 20(R) epimers were used, which limits direct comparison of activities across studies. Where specified in the original publications, we have indicated the epimer form.

#### 2.2.4. Molecular Weight and Membrane Permeability

The relationship between molecular weight and biological activity becomes particularly evident in the enhanced efficacy of deglycosylated ginsenosides. As the number of sugar moieties increases, the cytotoxic and anti-cancer activity of ginsenosides generally decreases [72]. This inverse relationship extends to immunomodulatory activities, where rare ginsenosides with fewer sugar groups demonstrate superior bioavailability and cellular uptake.

Low molecular weight contributes to easier membrane penetration, as compounds face an upper molecular weight limit for absorption across biological barriers [73]. This principle explains why rare ginsenosides like F1 and Rh1, with molecular weights around 638 Da, show enhanced cellular activities compared to their multi-glycosylated precursors. Ginsenoside Rb1 showed only 4.35% oral bioavailability in rats due to its large molecular weight (1109.46 Da) and poor membrane permeability [74].

#### 2.2.5. Receptor Interactions and Signal Transduction

Recent studies reveal specific receptor interactions that mediate the immunomodulatory effects of rare ginsenosides. Ginsenoside Rg5 specifically activates IGF-1R with an half maximal effective concentration (EC50) value of approximately 90 nM, promoting angiogenesis and vasorelaxation through multiple downstream signaling pathways [65]. This finding demonstrates that rare ginsenosides act as selective receptor modulators beyond their traditional anti-inflammatory roles.

The ability of ginsenosides to modulate multiple signaling pathways simultaneously appears to be structure-dependent. Ginsenoside Rg5 inhibits both the PI3K/AKT and MAPK pathways in LPS-stimulated microglia, while also suppressing reactive oxygen species (ROS) production through upregulation of HO-1 expression [64]. This multi-target approach is characteristic of rare ginsenosides with specific structural features, potentially explaining their superior therapeutic potential compared to major ginsenosides [75].

#### 2.2.6. Structure-Based Design Implications

The accumulated SAR data provide valuable insights for the rational design of ginsenoside-based immunomodulators. Key design principles include: selective deglycosylation at specific positions while maintaining critical sugar moieties, as demonstrated by F1’s NK cell activation requiring the C-20 glucose but not C-6 glycosylation [25]; optimization of stereochemistry at C-20 which significantly influences biological activities [70]; molecular weight reduction to enhance membrane permeability, explaining why rare ginsenosides exhibit superior bioavailability compared to their multi-glycosylated precursors [73]; and preservation of key hydroxyl groups that facilitate specific receptor binding, as demonstrated by Rg5’s insulin-like growth factor-1 receptor (IGF-1R) interaction [65].

## 3. Individual Ginsenoside Profiles: Unique Immunomodulatory Mechanisms

**Figure 2:**
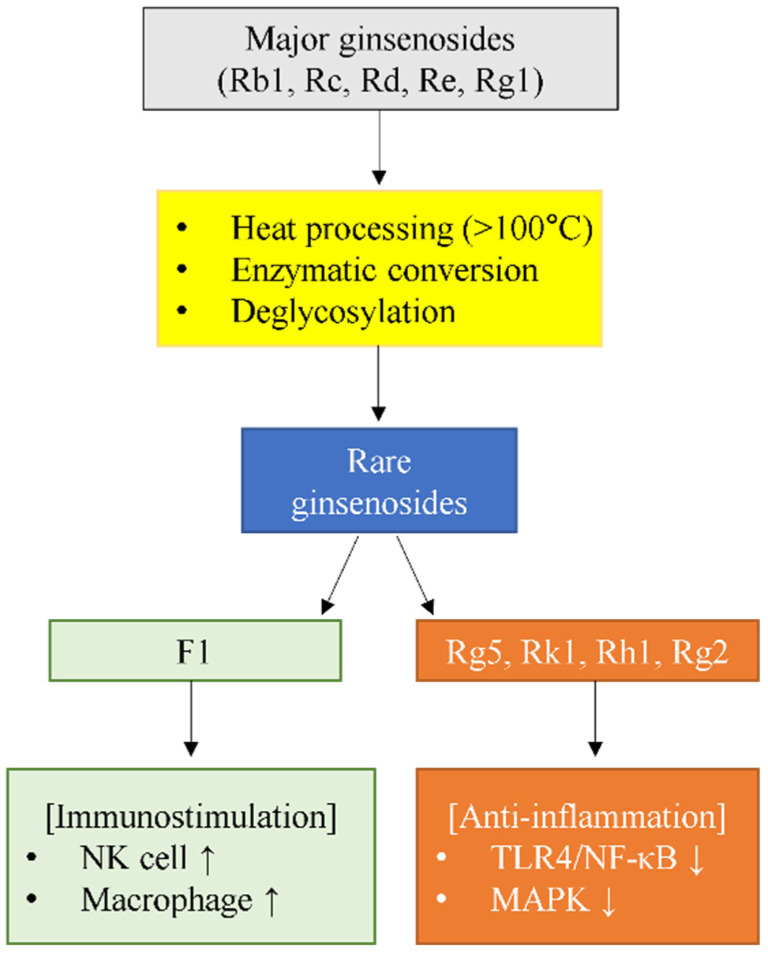
Production pathways and divergent immunomodulatory mechanisms of five rare ginsenosides. Major ginsenosides (Rb1, Rc, Rd, Re, Rg1) are transformed into rare ginsenosides through heat processing (>100 °C), enzymatic conversion, and deglycosylation. F1 uniquely stimulates immune responses by enhancing NK cell cytotoxicity and macrophage phagocytosis, while Rg5, Rk1, Rh1, and Rg2 suppress inflammatory responses through inhibition of TLR4/NF-κB and MAPK pathways. Arrows indicate the direction of immunomodulation (upward for stimulation, downward for suppression). NK, natural killer; TLR4, Toll-like receptor 4; NF-κB, nuclear factor-kappa B; MAPK, mitogen-activated protein kinase.

The five rare ginsenosides exhibit fundamentally divergent immunomodulatory effects that distinguish them not only from major ginsenosides but also from each other. F1 uniquely demonstrates immunostimulatory properties through NK cell activation and macrophage enhancement, contrasting sharply with Rg5, Rk1, Rh1, and Rg2, which primarily function as anti-inflammatory agents through distinct molecular mechanisms. This fundamental dichotomy—F1’s immune enhancement versus the others’ immune suppression—represents a critical distinction for therapeutic applications, as illustrated in Figure 2.

### 3.1. Ginsenoside F1: The Exceptional Immune Enhancer

#### 3.1.1. Unique Immunostimulatory Profile Among Rare Ginsenosides

Ginsenoside F1 stands out as the only immunostimulatory compound among 15 tested ginsenosides, uniquely enhancing rather than suppressing immune responses. This distinctive characteristic was demonstrated through systematic comparison where F1 at 10 μM concentration showed the most potent NK cell activation [25]. The differential regulation was further confirmed by F1’s ability to increase NK cell function without suppressing macrophage responses, contrasting with dexamethasone which potently suppressed macrophage activation at 2 mg/kg [27].

F1 is a metabolite of ginsenoside Re and Rg1, formed through intestinal bacterial transformation via stepwise deglycosylation, with Rh1 and F1 as the main metabolites [43]. The metabolic pathway follows Rg1 → Rh1 → F1 through sequential removal of sugar moieties at the C-6 position [25,43]. The enzymatic conversion can be achieved using commercial Cellulase KN from *Aspergillus niger*, enabling production of 13.0 g F1 from 50 g protopanaxatriol-type ginsenoside mixture substrate with 91.5 ± 1.1% purity [44].

SAR studies revealed the molecular basis for F1’s unique properties. The sugar moiety attached at C-20 is required for NK cell activation, whereas that at C-6 is dispensable, as demonstrated by comparative analysis where F1 with glucose at C-20 enhanced NK cell functions while Rh1 with glucose at C-6 did not [25]. F1-treated groups showed 1.5- to 2-fold higher frequency of NK1.1high cells compared to controls [63], with enhanced expression of degranulation marker cluster of differentiation (CD) 107a and increased intracellular calcium mobilization upon NK cell activation [25]. The unique immune enhancement contrasts with other ginsenosides’ effects, as F1 significantly attenuated eosinophilic inflammation through NK cell activation rather than broad immunosuppression [27].

#### 3.1.2. NK Cell Activation Mechanisms

The mechanism underlying F1’s unique immunostimulatory effect involves IGF-1-dependent NK cell activation. IGF-1 blockade antagonized NK cell potentiation by F1, while IGF-1 treatment alone recapitulated the effect. F1-treated NK cells showed enhanced phosphorylation of AKT rather than ERK, induced by co-engagement of natural killer group 2 member D (NKG2D) and CD244 receptors at 10 μM concentration, with calcium-mediated signaling modestly enhanced [25].

Downstream signaling cascades reveal F1’s distinct activation pattern. Enzymatic bioconversion of ginseng powder containing F1 resulted in 1.5- to 2-fold higher frequency of NK1.1^high^ cells in treated mice, with the PI3K-AKT-mammalian target of rapamycin (mTOR) pathway inducing granzyme B (GZMB) expression in NK cells. Mature NK cells produce high levels of interferon-gamma (IFN-γ) when stimulated via receptor activation [63]. This PI3K-AKT preference distinguishes F1 from other NK cell activators that primarily use MAPK pathways [76].

F1’s IGF-1/IGF1R pathway activation extends beyond NK cells to vascular effects. F1 promotes angiogenesis by enhancing endothelial cell proliferation, migration, and tube formation through PI3K/AKT/endothelial nitric oxide synthase (eNOS) signaling [26]. This dual immunostimulatory and angiogenic activity suggests therapeutic potential in conditions requiring both immune activation and vascular support [77,78,79].

#### 3.1.3. In Vivo Efficacy in Cancer and Inflammatory Models

In vivo efficacy studies have validated the mechanisms identified in vitro, demonstrating F1’s therapeutic potential across multiple disease models through NK cell-dependent mechanisms. In cancer immunosurveillance, F1 improved outcomes in mouse models of lymphoma clearance and metastatic melanoma that rely on NK cell activity, with F1 treatment significantly preventing lung colonization of systemically injected B16F10 cells following intraperitoneal administration [25]. F1 also reduced alpha-melanocyte stimulating hormone (α-MSH)-induced melanin secretion in B16F10 cells by 60% through inducing dendrite retraction via ras homolog (Rho) family guanosine triphosphatase (GTPase) modulation [80]. Using anti-asialo-monosialotetrahexosylganglioside 1 (GM1) antibody for NK cell depletion, the anti-tumor effects of F1 were completely abolished, confirming NK cell dependency [25]. Enzymatic bioconversion of ginseng powder containing F1 resulted in 1.5- to 2-fold higher frequency of NK1.1high cells in treated mice [63].

Beyond cancer, F1 demonstrated remarkable efficacy in neurodegenerative disease models. In amyloid precursor protein Swedish mutation (APPswe)/presenilin 1 deletion of exon 9 (PSEN1dE9) double-transgenic Alzheimer’s disease mice, 8-week oral administration of F1 jelly restored spatial working memory in the Y-maze task, though context-dependent fear memory remained unaffected [81]. F1 administration reduced Aβ plaque area and density specifically in the cortex but not in the hippocampus, with abnormally reduced phosphorylated cyclic adenosine monophosphate (cAMP) response element-binding protein (CREB) levels restored to normal and brain-derived neurotrophic factor (BDNF) augmented in the cortex [81]. These findings align with previous reports on Rg1, F1’s precursor, which showed neuroprotective effects through multiple pathways [77].

F1 also showed therapeutic efficacy in inflammatory disease models. In eosinophilic chronic rhinosinusitis mice, F1 administered at 50 mg/kg intraperitoneally or 3.5 mg/kg intranasally three times weekly significantly attenuated eosinophilic inflammation, with reduced IL-4 and IL-13 expression [27]. NK cell depletion nullified F1’s therapeutic effects but not dexamethasone’s, supporting a causal link between F1 and NK cell activity [27]. In apolipoprotein E (ApoE)^−/−^ atherosclerosis models, 8-week oral F1 treatment at 50 mg/kg/day induced remarkable reduction in atherosclerotic lesion area and decreased lectin-like oxidized low-density lipoprotein receptor-1 (LOX-1) and TLR4 expression [82]. Protein microarray demonstrated F1 significantly inhibited granulocyte colony-stimulating factor (G-CSF), intercellular adhesion molecule-1 (ICAM-1), macrophage inflammatory protein-1 delta (MIP-1δ), IL-1α, IL-15, and IL-16 levels through TNF-α-induced protein 3 (A20)-mediated suppression of NF-κB signaling [82].

#### 3.1.4. Bioavailability Enhancement and Clinical Applications

The clinical translation of F1 faces several challenges that are being addressed through advanced formulation strategies (detailed in Section 7.2). Poor oral bioavailability remains a major limitation, as ginsenosides including F1 exhibit high polarity and are subject to extensive gut microbiota-mediated biotransformation [83,84]. The major transport mechanism involves passive transcellular diffusion, with P-glycoprotein-mediated active efflux further limiting absorption [85]. Following oral administration of ginsenoside Rg1 at 25 mg/kg in rats, fecal excretion recoveries were 40.11% for Rg1, 22.19% for Rh1, and 22.88% for protopanaxatriol, while only 0.04% appeared in urine [83].

To overcome bioavailability challenges, nanostructured lipid carrier (NLC) formulations have been developed (see Section 7.3 for emerging formulation technologies). F1-loaded NLC achieved spherical particles averaging 98.9 nm with 90% encapsulation efficiency, increasing Caco-2 cell permeability to 39.2% versus 26.0% for free F1 [85]. Personalized bioconversion patterns have been identified, with *Bifidobacterium adolescentis* showing selectivity for 4-nitrophenyl-β-D-glucopyranoside substrates, while diet patterns significantly influence metabolite profiles between high-fat and low-fat dietary subjects [63].

Clinical applications have emerged across multiple indications. A cream containing 0.1% F1 showed significant whitening effects on artificially tanned human skin after 8 weeks, mediated by enhanced IL-13 production from epidermal gamma delta T (γδ T) cells [84]. In cardiovascular applications, SIRT1 activation by F1 protected against oxidative damage in cardiomyocytes, recovering oxygen consumption rate in tertiary-butyl hydroperoxide (t-BHP) injured cells [86]. Ginsenoside F1 attenuated pirarubicin-induced cardiotoxicity through Nrf2 and AKT/b-cell lymphoma 2 (Bcl-2) signaling pathways, reducing cytoplasmic cytochrome c levels and cleaved-caspase 3 expression [87]. For neurodegenerative diseases, 8-week oral administration of F1 jelly improved spatial working memory in APPswe/PSEN1dE9 transgenic mice, with reduced Aβ plaque density in the cortex and restored phosphorylated CREB levels [81]. F1 could pass the blood–brain barrier within 2 h after administration, protecting against amyloid beta-induced toxicity [24].

Safety profiles appear favorable, with F1 treatment in ApoE^−/−^ mice at 50 mg/kg/day for 8 weeks showing no adverse effects while reducing atherosclerotic lesions and inflammatory markers [82]. Future development requires optimization of delivery systems and standardization of gut microbiota-mediated metabolism for consistent therapeutic outcomes.

### 3.2. Ginsenoside Rg5: Direct TLR4 Antagonist

#### 3.2.1. TLR4-Mediated Anti-Inflammatory Mechanisms

Ginsenoside Rg5 distinguishes itself among rare ginsenosides through its direct interaction with TLR4, a pattern recognition receptor crucial for innate immunity [88]. Unlike other ginsenosides that modulate inflammation through indirect pathways, Rg5 specifically blocks the binding of LPS to TLR4 on macrophages, thereby preventing the initial step of inflammatory cascade activation [88,89]. This direct receptor antagonism has been demonstrated in multiple cell types including macrophages and microglial cells [64,89].

Rg5 demonstrates potent anti-inflammatory effects through multiple interconnected pathways. In LPS-stimulated BV2 microglial cells, Rg5 significantly suppressed nitric oxide production and reduced proinflammatory TNF-α secretion in a dose-dependent manner [64]. The compound also inhibited the mRNA expressions of inducible nitric oxide synthase (iNOS), TNF-α, IL-1β, cyclooxygenase (COX)-2, and matrix metalloproteinase (MMP)-9 induced by LPS in both microglial cells and macrophages [64,90].

Mechanistically, Rg5 inhibits the phosphorylation of PI3K/AKT and all three MAPKs (ERK, c-jun n-terminal kinase (JNK), and p38), effectively blocking upstream inflammatory signaling [64,91]. The compound suppresses the DNA binding activities of both NF-κB and AP-1, which are key transcription factors controlling inflammatory reactions [92,93]. Additionally, Rg5 upregulates HO-1 expression while suppressing ROS production, contributing to its antioxidant effects through the Nrf2 pathway [64,94].

#### 3.2.2. HMGB1-Mediated Septic Response Inhibition

A distinctive feature of Rg5 is its ability to suppress HMGB1-mediated SIRT1 activation [66]. Rg5 treatment enhanced SIRT1-mediated deacetylation of HMGB1, preventing its release from activated immune cells [61,66]. In cecal ligation and puncture-induced sepsis models, Rg5 administration (20 mg/kg) resulted in 70% survival rate compared to 20% in control groups [66].

The compound demonstrated remarkable efficacy in reducing inflammatory mediator production, including TNF-α and IL-6, through inhibition of NF-κB and ERK1/2 activation [61]. These protective effects were associated with decreased tissue inflammation and preserved organ function in septic conditions, as documented in comprehensive pharmacological reviews [95].

Beyond sepsis, Rg5 exhibits organ-protective effects in various inflammatory conditions. In acetaminophen-induced liver injury models, Rg5 suppressed the expression of inflammatory cytokines TNF-α and IL-1β, providing hepatoprotective effects [96]. In hyperuricemia nephropathy model induced by yeast extract and adenine, Rg5 reduced serum uric acid, blood urea nitrogen (BUN), and creatinine levels while inhibiting nicotinamide adenine dinucleotide phosphate (NADPH) oxidase 1 (NOX1) expression, thereby suppressing the TLR4 pathway and alleviating oxidative stress, inflammation, pyroptosis, and apoptosis [97]. The anti-inflammatory activity of Rg5 was found to be superior compared to ginsenosides Rb1, Rd and Rg3, which was hypothesized to be due to its higher lipophilicity [98].

#### 3.2.3. Synergistic Anti-Inflammatory Effects

Rg5 exhibits synergistic anti-inflammatory effects when combined with its structural isomer Rk1 [66,99], demonstrated through direct co-administration studies. The combination of Rg5 and Rk1 showed enhanced suppression of HMGB1-mediated inflammatory responses compared to individual compounds [61]. This synergy was particularly evident in reducing proinflammatory cytokine production and improving survival outcomes in sepsis models [93,95].

The synergistic mechanism involves complementary actions on different signaling pathways, with Rg5 primarily targeting TLR4/NF-κB while Rk1 modulates SIRT1 and other inflammatory mediators [93,95]. Recent studies have shown that the Rg5/Rk1 combination also affects gamma-aminobutyric acid (GABA)-ergic/serotoninergic signaling pathways and stimulates osteoblast proliferation, suggesting broader therapeutic applications [99,100].

The Rg5/Rk1 combination demonstrates remarkable effects on bone metabolism and osteoporosis prevention. Studies showed that Rg5/Rk1 stimulates osteoblast cell growth and promotes the expression of osteoblastic markers including alkaline phosphatase activity, type I collagen content, bone morphogenetic protein-2 (BMP-2) and calcium deposition in dose-dependent manners [101]. The combination stimulates mRNA expression of runt-related transcription factor 2 (Runx2) and osteocalcin, indicating its potential to prevent osteoporosis through the BMP-2/Runx2 signaling pathway [101].

#### 3.2.4. Additional Activities and Clinical Translation

Beyond its anti-inflammatory properties, Rg5 demonstrates diverse biological activities through distinct mechanisms. The compound specifically activates IGF-1R with an EC50 value of ~90 nM, promoting angiogenesis and vasorelaxation [65]. This IGF-1R activation leads to enhanced endothelial cell proliferation, migration, and tube formation through PI3K/AKT/eNOS and other downstream signaling pathways [65,102].

In metabolic disease models, Rg5 ameliorates insulin resistance by modulating gut microbiota composition and reducing metabolic endotoxemia. The compound significantly decreased the Firmicutes/Bacteroidetes ratio and restored intestinal barrier function, leading to reduced LPS translocation and systemic inflammation [103]. Additionally, Rg5 inhibited succinate-associated lipolysis in adipose tissue through suppression of endoplasmic reticulum (ER) stress and nucleotide-binding oligomerization domain-like receptor protein 3 (NLRP3) inflammasome activation [104].

The therapeutic potential of Rg5 is supported by its favorable safety profile and tissue distribution patterns. Pharmacokinetic studies indicate that Rg5 achieves the highest exposure level in lung tissue, which may explain its efficacy in respiratory inflammatory conditions [28,91]. The compound has also shown promise in neuroinflammatory conditions, protecting against glutamate-induced neurotoxicity in Huntington’s disease models [105].

Production methods for Rg5 have been optimized through controlled steaming processes of ginseng, with various heating conditions affecting the conversion efficiency from major ginsenosides [46,47]. Recent advances in biotransformation technologies using enzymatic conversion and heat-induced deglycosylation have improved production yields [10]. The stability and bioavailability of Rg5 make it a promising candidate for clinical applications in inflammatory diseases [93,106].

### 3.3. Ginsenoside Rk1: Multi-Pathway Modulator

#### 3.3.1. Broadest Pathway Coverage Among Rare Ginsenosides

Ginsenoside Rk1 distinguishes itself among rare ginsenosides through its exceptionally broad spectrum of molecular targets [107]. As a dehydrated derivative of ginsenoside Rg3 formed during heat processing at 120 °C, Rk1 demonstrates the most comprehensive pathway modulation capabilities in this compound class [108].

The compound’s pharmacological profile encompasses simultaneous inhibition of three major inflammatory pathways. Rk1 suppresses NF-κB signaling through reduced p65 nuclear translocation [90]. In HMGB1-mediated septic responses, Rk1 and Rg5 treatment (20 mg/kg) achieved 70% survival rate compared to 20% in controls, with significant TNF-α and IL-6 reduction [66]. Rk1 also inhibits p38 MAPK phosphorylation at Ser505 in platelet activation [109] and modulates ERK/JNK/p38 pathways in epithelial–mesenchymal transition [108].

Beyond primary targets, Rk1 affects multiple signaling cascades. The compound suppresses PI3K/AKT/NF-κB signaling in ultraviolet B (UVB)-irradiated keratinocytes [110] and regulates glutamine metabolism through ERK/c-Myc inhibition in hepatocellular carcinoma [111]. Rk1 also demonstrates protective effects on endothelial barrier function, inhibiting vascular endothelial growth factor (VEGF)-induced permeability through tight junction stabilization and cortical actin ring formation [112].

Systematic review of 28 studies confirmed Rk1’s multi-pathway modulation, including anticancer effects half maximal inhibitory concentration (IC50) 70 μM in A549 cells), COX-1/2 inhibition, and nephroprotective properties [22,107,113]. This broad mechanistic coverage positions Rk1 as a promising therapeutic candidate for complex diseases requiring multi-target intervention.

#### 3.3.2. Triple Pathway Inhibition: NF-κB, p38 MAPK, and STAT Signaling

The therapeutic efficacy of ginsenoside Rk1 is fundamentally linked to its ability to simultaneously inhibit three critical inflammatory signaling pathways that often exhibit cross-talk in pathological conditions [107,114]. Rk1 significantly suppresses NF-κB transcriptional activity through prevention of inhibitor of nuclear factor kappa B alpha (IκBα) phosphorylation and p65 nuclear translocation, as demonstrated in HMGB1-mediated septic models where treatment (20 mg/kg) reduced pro-inflammatory cytokines TNF-α and IL-6 [66,90,110]. In UVB-irradiated keratinocytes and LPS-stimulated macrophages, Rk1 downregulated NF-κB signaling achieving up to 90% inhibition at 25 μM [90,110].

Parallel to NF-κB suppression, Rk1 demonstrates potent inhibition of MAPK signaling cascades. The compound dose-dependently suppresses p38 phosphorylation at Ser505, subsequently affecting downstream targets including cytosolic phospholipase A2 (cPLA2)-mediated thromboxane A2 production in collagen-induced platelet activation [109]. In transforming growth factor beta 1 (TGF-β1)-induced epithelial–mesenchymal transition models, Rk1 suppressed not only p38 but also ERK and JNK phosphorylation, indicating broad MAPK pathway modulation [108]. Additionally, Rk1 blocks Janus kinase (JAK) 2/STAT3 activation in myocardial ischemia models and reduces STAT1 signaling in TNF-α/IFN-γ-stimulated cells [90,110].

By inhibiting these three pathways concurrently, Rk1 achieves more comprehensive inflammatory suppression than single-pathway-targeting approaches. The simultaneous modulation of NF-κB, MAPK, and JAK/STAT pathways disrupts their interconnected signaling networks, preventing compensatory activation mechanisms that often limit the efficacy of single-target therapies [106,114].

#### 3.3.3. Additional Therapeutic Targets

Beyond its triple pathway inhibition, Rk1 demonstrates additional therapeutic mechanisms that distinguish it from other rare ginsenosides. The compound exhibits protective effects through PI3K/AKT signaling, particularly in oxidative stress conditions. In H_2_O_2_-induced melanocyte injury, Rk1 (0.1–0.4 mM) significantly improved cell viability and reduced apoptotic rate through PI3K/AKT/Nrf2/HO-1 pathway activation, with PI3K inhibitor LY294002 reversing these protective effects [115]. Similarly, Rk1 prevented UVB irradiation-mediated oxidative stress, inflammatory response, and collagen degradation via PI3K/AKT/NF-κB pathway in vitro and in vivo [110].

A distinctive feature of Rk1 is its remarkable ability to protect vascular barriers. In human retinal endothelial cells, Rk1 strongly inhibited permeability induced by VEGF, advanced glycation end-products, thrombin, and histamine, while significantly reducing vessel leakiness in diabetic mouse retina [112]. This anti-permeability activity correlated with enhanced stability and positioning of tight junction proteins zonula occludens-1 (ZO-1) and occludin at cell boundaries, achieved through Rk1-induced phosphorylation of myosin light chain and cortactin, leading to cortical actin ring formation [112]. The compound also suppressed platelet-mediated thrombus formation by downregulating granule release and integrin alpha IIb beta 3 (αIIbβ3) activation [109], with 10 μM Rk1 decreasing thromboxane B2 levels by 77% in washed platelets [113].

Additional mechanisms include COX enzyme inhibition and neuroprotection. At 20 μM concentration, Rk1-derived inhibition was higher on COX-2 than on COX-1 [113]. The compound demonstrated neuroprotective effects in Parkinson’s disease models, alleviating 1-methyl-4-phenyl-1,2,3,6-tetrahydropyridine (MPTP)-induced motor deficits and preserving tyrosine hydroxylase expression in substantia nigra through SIRT3-mediated Nrf2/HO-1 signaling pathway [116]. In hepatocellular carcinoma cells, Rk1 regulated glutamine metabolism through inhibition of ERK/c-Myc pathway, suppressing glutaminase 1 (GLS1) expression and inducing G0/G1 cell cycle arrest [111]. Recent development of Rk1-modified liposomes achieved 97.24 ± 0.75% encapsulation efficiency with enhanced tumor targeting capabilities [117].

#### 3.3.4. Clinical Development and Combination Strategies

Pharmacokinetic studies in rats revealed Rk1’s moderate half-life (3.09–3.40 h), time to maximum concentration of 4.29–4.57 h, and low oral bioavailability (2.87–4.23%) (formulation solutions discussed in Section 7.2) [118]. The clinical translation of Rk1 has advanced through multiple therapeutic strategies and formulation approaches. The synergistic effects with Rg5 have been particularly promising, with combined Rg5/Rk1 treatment showing enhanced suppression of HMGB1-mediated inflammatory responses and improved survival outcomes in sepsis models compared to individual compounds [107]. This synergy was demonstrated in osteoblast proliferation studies where the 1:1 ratio of Rg5/Rk1 showed optimal mineralization activity [119]. The combination also affects GABAergic/serotoninergic signaling pathways, suggesting broader therapeutic applications [107].

Novel drug delivery systems have significantly improved Rk1’s therapeutic potential. Rk1-loaded liposomes modified with ginsenoside structure achieved 97.24% encapsulation efficiency and enhanced tumor targeting through glucose transporter 1 (GLUT1) protein binding [117]. The formulation reduced tumor volume by over 50% compared to conventional cholesterol-containing liposomes while simultaneously relieving *Candida albicans* infection burden in immunocompromised cancer patients [117]. γ-cyclodextrin inclusion complexes enhanced the dissolution rate of Rk1, with one tablet releasing equivalent amounts to capsules containing 2.2–2.3 times greater doses of the raw extract [120].

Rk1 demonstrates broad combination therapy potential across multiple disease models. In platelet aggregation studies, Rk1 at 10 μM showed stronger anti-platelet effects than aspirin at 100 μM [113]. The compound suppressed platelet aggregation through inhibition of cPLA2 phosphorylation and reduced thromboxane A2 generation in a dose-dependent manner [109]. Rk1 demonstrated anti-cancer effects through inhibition of glutamine metabolism via the ERK/cellular myelocytomatosis oncogene (c-Myc) pathway, significantly reducing adenosine triphosphate (ATP) production in HepG2 and LM3 cells [111].

Safety profiles support clinical development, with Rk1 demonstrating protective effects against chemotherapy-induced toxicities. In cisplatin nephrotoxicity models, steamed Vietnamese ginseng containing Rk1 showed kidney-protective effects with half maximal response concentration (RC50) values of 46.15–88.4 μM for S-form ginsenosides [121]. Rk1 protected human melanocytes from H_2_O_2_-induced oxidative injury by regulating the PI3K/AKT/Nrf2/HO-1 pathway, with 0.4 mM providing optimal protection without cytotoxicity [115]. In hepatotoxicity studies, Rk1 pretreatment at 10–20 mg/kg significantly reduced serum alanine aminotransferase (ALT) and aspartate aminotransferase (AST) levels in acetaminophen-induced liver injury [122].

Production optimization has facilitated clinical translation prospects. Heat processing at 120 °C for 3–12 h converted major ginsenosides to Rk1 with yields reaching maximum at 12 h [121]. Consecutive high-speed counter-current chromatography successfully separated Rk1 with high purity from black ginseng using n-hexane/ethyl acetate/methanol/water solvent systems [123]. These standardized production methods ensure consistent quality for clinical applications, with Rk1 content reaching 1.0% in standardized red ginseng preparations [120].

### 3.4. Ginsenoside Rh1: Anti-Allergic Specialist

#### 3.4.1. Unique Anti-Allergic Profile Among Rare Ginsenosides

Ginsenoside Rh1 stands out as the most potent anti-allergic compound among rare ginsenosides, demonstrating superior efficacy to conventional anti-allergic drugs When tested at 25 mg/kg in mice, Rh1 achieved 87% inhibition of immunoglobulin (Ig) E-mediated passive cutaneous anaphylaxis, significantly exceeding the 31% inhibition observed with disodium cromoglycate at the same dose. This enhanced efficacy stems from its membrane-stabilizing action, as confirmed by differential scanning calorimetry [124].

The anti-allergic effects of Rh1 extend across multiple disease models. In atopic dermatitis models, oral Rh1 administration at 20 mg/kg reduced oxazolone-induced skin lesions in hairless mice while decreasing serum IgE and IL-6 levels [125]. Its metabolite, PPT, further contributes to therapeutic efficacy by suppressing trinitrobenzenesulfonic acid (TNBS)-induced colitis in mice at 20 mg/kg dose [126]. This metabolite also demonstrates direct anti-inflammatory activity through inhibition of histamine release and PGE (prostaglandin E) 2 production in IgE-mediated mast cell activation [127].

A comprehensive analysis of Rh1’s mechanisms reveals multiple pathways contributing to its anti-allergic effects. Systematic review of 57 studies has established that Rh1 exhibits anti-inflammatory, antioxidant, and immunomodulatory effects [41]. Recent mechanistic investigations have uncovered that Rh1 alleviates allergic rhinitis through adenosine monophosphate-activated protein kinase (AMPK)/unc-51-like autophagy activating kinase 1 (ULK1)/FUN14 domain-containing 1 (FUNDC1)-mediated mitophagy, reducing NLRP3 inflammasome activation in human nasal epithelial cells [128]. Additionally, protopanaxatriol-type ginsenosides, including Rg1 and 20(S)-Rg3, have been shown to induce IgA production by mouse B cells, suggesting immunomodulatory effects that complement the direct anti-allergic actions [129].

The therapeutic potential of Rh1 is significantly enhanced when combined with glucocorticoids. Rh1 potentiates dexamethasone’s anti-inflammatory effects in chronic inflammatory disease models by reversing dexamethasone-induced resistance [130]. This synergistic interaction was further validated in Murphy Roths Large/lymphoproliferation (MRL/lpr) mice, where Rh1 combined with dexamethasone improved autoantibody production and lymphoproliferation [131]. At the molecular level, Rh1 upregulates phase II antioxidant enzymes including HO-1 through Nrf2/antioxidant response element (ARE) signaling in rat primary astrocytes [132]. Long-term studies have demonstrated that Rh1 administration enhances learning and memory by promoting cell survival in mouse hippocampus [133]. Collectively, these findings position metabolite ginsenosides including Rh1 as more potent suppressors of allergic reactions compared to parent ginsenosides Rb1 and Re [134].

#### 3.4.2. Mast Cell Stabilization and Histamine Release Inhibition

While Rh1 modulates T cell responses as described above, its effects on innate immune cells, particularly mast cells, provide complementary mechanisms for allergic disease control. Ginsenoside Rh1 exhibits potent mast cell stabilizing activity through direct membrane interactions. In rat peritoneal mast cells activated by compound 48/80, Rh1 significantly inhibited histamine release with superior efficacy compared to standard anti-allergic drugs. The membrane-stabilizing properties were directly confirmed using differential scanning calorimetry, revealing Rh1’s interaction with mast cell membrane lipids. This translates to remarkable in vivo efficacy, with Rh1 at 25 mg/kg achieving 87% inhibition in passive cutaneous anaphylaxis (PCA) models, markedly exceeding disodium cromoglycate’s 31% inhibition at the same dose [124].

The inhibitory effects extend to various mast cell-derived mediators. Korean red ginseng and its ginsenosides, particularly Rh2, have been shown to suppress β-hexosaminidase release from RBL-2H3 cells stimulated with IgE-antigen complexes [124]. Similar protective effects emerge from fermented red ginseng containing Rh1 metabolites, which not only inhibited cellular degranulation but also reduced compound 48/80-induced scratching behavior by 45–47% in mice [135]. Further supporting these findings, ginseng extracts containing protopanaxatriol ginsenosides demonstrated approximately 30% reduction in histamine release from rat mast cells, with cell viability maintained above 80% at 0.5 mg/mL [136].

The molecular mechanisms underlying these effects have been elucidated through studies on PPT, a common metabolite of protopanaxatriol-type ginsenosides including Rh1. PPT inhibited calcium influx and protein kinase C (PKC) activation, suppressed spleen tyrosine kinase (Syk) protein expression and downstream signaling through phospholipase Cγ (PLCγ) and phospholipase D (PLD) pathways, and reduced leukotriene synthesis through inhibition of phospholipase A2 (PLA2) and COX-1/2 expression [127]. These findings suggest that Rh1 likely employs similar mechanisms for its direct mast cell stabilizing effects.

The anti-inflammatory effects complement the mast cell stabilizing properties, as Rh1 inhibited iNOS and COX-2 protein expression while suppressing NF-κB activation in RAW264.7 macrophages [124]. This creates a synergistic therapeutic approach where Rh1 simultaneously shifts T helper (Th) 1/Th2 balance and stabilizes mast cells. The dual mechanism results in reduced Th2 cytokines (IL-4, IL-13) that decrease IgE production and mast cell priming [137], while direct mast cell stabilization prevents acute degranulation. These complementary pathways converge to provide comprehensive allergic disease control, as will be further demonstrated in the clinical studies discussed in Section 3.4.3.

#### 3.4.3. Modulation of Th2-Mediated Allergic Responses

Ginsenoside Rh1 demonstrates potent modulatory effects on Th2-mediated immune responses, which are central to allergic inflammation. In ovalbumin-induced asthmatic mice, Rh1 treatment at 100 mg/kg comprehensively rebalanced the immune response by significantly suppressing the elevated levels of Th2 cytokines, including IL-4, IL-5, and IL-13 in both bronchoalveolar lavage fluid and serum. Concurrently, the treatment enhanced Th1 cytokines IL-12 and IFN-γ, indicating a restoration of Th1/Th2 balance that had been skewed toward Th2 dominance in allergic conditions [138].

This shift from Th2 to Th1 polarization occurs through multiple cellular mechanisms. In allergic rhinitis models, Rh1 administration significantly decreased IL-4-positive CD4+ T cells while increasing IFN-γ-positive CD4+ T cells in nasal-associated lymphoid tissue. The underlying molecular pathway involves AMPK/ULK1/FUNDC1-mediated regulation of mitochondrial autophagy, which suppresses NLRP3 inflammasome activation, thereby reducing IL-4 and IL-13 production in nasal lavage fluid [128].

Beyond cytokine modulation, Rh1 targets upstream regulatory elements of Th2 responses. Treatment with Rh1 significantly reduced IL-33 levels, an epithelial-derived alarmin that initiates and amplifies Th2 responses [138]. In atopic dermatitis models, oral Rh1 administration at 20 mg/kg promoted a regulatory phenotype by increasing forkhead box P3 (Foxp3) mRNA expression in draining lymph nodes while decreasing IL-4 expression, suggesting dual action through both Th2 suppression and regulatory T cell enhancement. This elevation of Foxp3+ regulatory T cells provides an additional mechanism for controlling excessive Th2 immunity [125].

The therapeutic consequences of Th2 suppression manifest as reduced tissue eosinophilia, a hallmark of allergic diseases. The decreased IL-5 production directly impacts eosinophil survival and activation, resulting in significantly reduced eosinophil counts in bronchoalveolar lavage fluid of asthmatic mice [138]. Furthermore, reduced IL-13 levels contribute to decreased mucus hypersecretion and airway remodeling, key features of chronic allergic inflammation [128]. Rh1 also targets eosinophil recruitment by suppressing eotaxin expression, the primary chemokine responsible for eosinophil migration to inflammatory sites, through inhibition of NF-κB activation [124]. This dual targeting—reducing both survival signals (IL-5) and recruitment signals (eotaxin)—combined with suppression of IgE-mediated responses, provides comprehensive control of tissue eosinophilia and allergic inflammation [124,125].

The molecular basis for these effects involves coordinated modulation of multiple signaling pathways. A systematic review of 57 studies confirmed that Rh1 modulates inflammatory responses through various mechanisms including NF-κB inhibition [41]. In inflammatory cells, Rh1’s suppression of NF-κB activation and COX-2 expression serves as upstream regulators of Th2 cytokine production [124]. Additionally, activation of MAP kinase pathways and Nrf2/ARE signaling contributes to an anti-inflammatory phenotype through upregulation of antioxidant enzymes that reduce oxidative stress-induced Th2 responses [132].

The immunomodulatory effects of Rh1 extend beyond simple Th2 suppression to encompass active immune regulation. In colitis models, Rh1 and its metabolite PPT not only suppressed inflammation but actively induced IL-10 production and promoted anti-inflammatory responses [126]. This combination of increased IL-10, enhanced Foxp3+ regulatory T cells, and restored Th1/Th2 balance creates a comprehensive immunoregulatory environment that effectively controls allergic inflammation [125,126]. These integrated mechanisms—encompassing Th2 cytokine suppression, Th1 enhancement, regulatory T cell induction, and consequent eosinophil reduction—establish Rh1’s potential as a multi-targeted therapeutic agent for Th2-mediated allergic diseases [41,138].

#### 3.4.4. Pharmaceutical Optimization and Safety Profile

The clinical translation of ginsenoside Rh1 has advanced significantly through pharmaceutical optimization and mechanistic validation. Self-microemulsifying drug delivery systems (SMEDDS) incorporating both cytochrome P450 (CYP450) and P-glycoprotein (P-gp) inhibitory excipients have transformed Rh1’s pharmacokinetic profile, achieving 33.25% oral bioavailability compared to 12.92% for the free drug—a 2.6-fold improvement that addresses the primary limitation of poor absorption [139]. Complementary transdermal approaches using transfersomes demonstrated superior skin permeation with 45–65% entrapment efficiency and particle sizes ranging from −20.95 to −31.37 mV zeta potential, enabling non-invasive delivery routes [140].

Drug interaction profiles reveal both challenges and therapeutic opportunities. Rh1 moderately inhibits cytochrome P450 3A4 (CYP3A4) with an IC50 of 76.9 ± 6.8 μM through competitive inhibition of testosterone 6β-hydroxylation, necessitating careful consideration in polypharmacy settings [41]. However, this interaction profile creates unique therapeutic advantages, as Rh1 potentiates dexamethasone’s anti-inflammatory effects while reversing glucocorticoid resistance in chronic inflammatory diseases. In collagen-induced arthritis models, the combination of Rh1 with dexamethasone enhanced glucocorticoid receptor expression and improved anti-inflammatory outcomes even after prolonged steroid treatment [130].

Comprehensive safety evaluations establish a robust foundation for clinical development. In acetaminophen-induced hepatotoxicity models, Rh1 at 30 mg/kg significantly enhanced hepatic antioxidant defenses by increasing glutathione (GSH) and superoxide dismutase (SOD) activities while reducing malondialdehyde (MDA) levels, AST, and ALT, demonstrating hepatoprotective rather than hepatotoxic effects [141]. Nephroprotective properties emerged in cisplatin-induced kidney injury studies, where Rh1 prevented HK-2 cell apoptosis through dual mechanisms: inhibiting ROS production and suppressing JNK and p53 pathway activation, with concurrent reduction in the kidney injury marker neutrophil gelatinase-associated lipocalin (NGAL) [142].

Mechanistic studies have identified specific molecular targets that define therapeutic applications. In allergic rhinitis models, Rh1’s therapeutic efficacy operates through activation of the AMPK/ULK1/FUNDC1 pathway, which mediates mitochondrial autophagy and subsequently reduces NLRP3 inflammasome activation while restoring Th1/Th2 balance [128]. For cardiovascular applications, Rh1 functions as a novel SIRT3 activator, mitigating mitochondrial dysfunction in myocardial ischemia through enhanced mitochondrial biogenesis and reduced oxidative stress, positioning it as a promising candidate for ischemic heart disease treatment [143].

Neurological applications demonstrate particular promise through multiple complementary mechanisms. Rh1 improved cognitive performance in scopolamine-induced amnesia models via dual pathways: upregulating memory-associated immediate early genes including CREB, early growth response 1 (Egr-1), c-Fos proto-oncogene (c-Fos), and cellular Jun proto-oncogen (c-Jun) in the hippocampus, while simultaneously enhancing cholinergic function through decreased acetylcholinesterase activity and increased acetylcholine levels [144]. In cerebral ischemia–reperfusion injury, seven days of Rh1 administration produced comprehensive neuroprotection, as evidenced by significantly improved neurological function scores, increased neuronal nuclei (NeuN)-positive neurons, elevated neurotrophic factors BDNF and nerve growth factor (NGF), and reduced oxidative stress markers—collectively demonstrating multi-target neuroprotective efficacy [145].

### 3.5. Ginsenoside Rg2: Dual-Compartment Immunomodulator

#### 3.5.1. Neuroprotective Mechanisms in Central Nervous System

Ginsenoside Rg2, a protopanaxatriol-type saponin with sugar moieties on the C-6 position, exhibits distinctive central nervous system neuroprotective properties through multiple convergent molecular pathways [146]. This compound’s selective therapeutic efficacy in neuronal protection emerges from its ability to target excitotoxicity, oxidative stress, and apoptotic cascades that characterize various neurodegenerative and neuropsychiatric disorders.

In glutamate excitotoxicity models, Rg2 treatment provides robust cytoprotection in PC12 neuronal cells through coordinated cellular defense mechanisms. The compound effectively reduces intracellular calcium accumulation, decreases malondialdehyde and nitric oxide production, and enhances endogenous superoxide dismutase activity. These protective mechanisms converge through downregulation of key pro-apoptotic mediators including caspase-3 and calpain II, thereby preventing glutamate-induced neuronal death [147].

Central nervous system protection by Rg2 extends beyond acute excitotoxicity to chronic neurodegenerative processes. In vascular dementia models, systemic administration at 5 mg/kg significantly preserves both neuronal integrity and cognitive function through multi-level interventions. The compound exerts potent anti-apoptotic effects within brain tissue by suppressing caspase-3 mRNA expression while simultaneously modulating the balance between pro-apoptotic and anti-apoptotic proteins—reducing bcl-2-associated x protein (Bax) and p53 protein levels while enhancing expression of neuroprotective proteins Bcl-2 and heat shock protein 70 (HSP70) [148].

Recent investigations have revealed additional neuroprotective mechanisms involving cellular quality control systems. Rg2 promotes neuroprotection through autophagy induction, activating this cellular clearance mechanism in an AMPK-ULK1-dependent and mTOR-independent manner to enhance removal of protein aggregates and improve cognitive behaviors in mouse models [149]. Furthermore, the compound’s protective effects extend to the neurovascular unit, where it protects blood–brain barrier integrity by alleviating astrocyte inflammation and reducing barrier permeability in neurodegeneration models [150].

At the neurotransmitter level, the compound modulates central neurotransmitter systems through direct interaction with human neuronal nicotinic acetylcholine receptors, regulating receptor-mediated calcium and sodium influx [151]. This receptor modulation translates to behavioral improvements, as Rg2 demonstrates efficacy in stress-related central nervous system dysfunction, exhibiting significant antidepressant-like effects in chronic mild stress models [152]. In cognitive impairment studies, the therapeutic benefits involve lysosomal pathway modulation, with Rg2 treatment ameliorating scopolamine-induced memory deficits through enhanced lysosomal function [153].

#### 3.5.2. Peripheral Organ Protection and Systemic Therapeutic Effects

Ginsenoside Rg2 demonstrates potent peripheral anti-inflammatory activities through targeted suppression of inflammatory signaling cascades, resulting in comprehensive organ protection across multiple tissue systems [154,155,156]. The compound’s anti-inflammatory mechanisms provide the molecular foundation for its diverse therapeutic applications in peripheral inflammatory conditions.

In inflammatory signaling modulation, Rg2 effectively suppresses key pro-inflammatory pathways with particular efficacy against the NF-κB/NLRP3 inflammasome axis. Treatment significantly decreased phosphorylation levels of inhibitor of nuclear factor kappa b kinase subunit beta (IKKβ), inhibitor of nuclear factor kappa b alpha (IκBα), and NF-κB p65 while inhibiting NLRP3 inflammasome activation through reduced expression of NLRP3, apoptosis-associated speck-like protein containing a caspase recruitment domain (ASC), and caspase-1 components. These molecular changes translate to substantial reductions in inflammatory cytokine release, including decreased production of IL-18 and IL-1β [154].

Cardiovascular inflammatory responses are markedly attenuated through distinct but complementary mechanisms. The compound effectively inhibited TNF-α, IL-1β, and IL-6 levels in myocardial ischemia–reperfusion models while suppressing necroptosis through transforming growth factor-beta-activated kinase 1 (TAK1) phosphorylation regulation. Additionally, preservation of tissue integrity was demonstrated through reduced lactate dehydrogenase release and Evans blue dye penetration, indicating that anti-inflammatory mechanisms directly contribute to myocardial protection [155].

Hepatic anti-inflammatory activity translates into significant protection against fibrotic progression. Rg2 treatment effectively reduced plasma lipopolysaccharide levels and inhibited pro-inflammatory transforming growth factor-beta 1 (TGF-β1) signaling while suppressing fibrotic markers including alpha-smooth muscle actin (α-SMA) and collagen type I alpha 1 (COL1A1) expression. The underlying mechanism involves AKT/mTOR pathway activation and autophagy regulation, creating a coordinated anti-fibrotic response [156].

Systemic anti-inflammatory effects extend beyond individual organs through multiple integrated pathways. In acute inflammatory conditions, the combination of Rg2 with ginsenoside Rh1 synergistically attenuated lipopolysaccharide-induced organ damage through suppression of TLR4-STAT1 activation and reduced inflammatory cytokine production in macrophages [42]. The compound also exhibits prebiotic anti-inflammatory properties by promoting growth of Parabacteroides distasonis, which contributes to systemic anti-inflammatory effects through modulation of secondary bile acid production and macrophage M2 polarization [157].

Additional therapeutic mechanisms include regulation of bone remodeling, where the compound demonstrates anti-inflammatory efficacy by inhibiting receptor activator of nuclear factor kappa-B ligand (RANKL)-induced inflammatory osteoclastogenesis through suppression of nuclear factor of activated T-cells, cytoplasmic 1 (NFATc1), c-Fos, and MAPK signaling pathways [31]. These diverse anti-inflammatory mechanisms—spanning from molecular signaling to microbiome modulation—collectively establish Rg2 as a multi-target peripheral immunomodulator with significant therapeutic potential across inflammatory disease conditions.

#### 3.5.3. Anti-Inflammatory and Immune Modulation

Ginsenoside Rg2 demonstrates potent anti-inflammatory and immunomodulatory effects through multiple molecular pathways, positioning it as a promising therapeutic agent for inflammatory diseases. The compound’s efficacy stems from its ability to target key inflammatory signaling cascades, including the TLR4-STAT1 and NF-κB pathways, while effectively reducing pro-inflammatory cytokine production and tissue damage.

Rg2’s primary anti-inflammatory mechanism involves direct interference with TLR4-mediated inflammatory cascade. In macrophages, the combination of rare ginsenosides Rg2 and Rh1 significantly decreased LPS-induced major inflammatory mediator production, inducible-nitric oxide synthase expression, and nitric oxide production. This suppression occurs through inhibition of LPS binding to TLR4 on peritoneal macrophages, thereby blocking the initial step of TLR4-mediated signaling pathway. In vivo validation demonstrated that treatment with 20 mg/kg ginsenoside significantly reduced LPS-induced acute tissue inflammation levels, as evidenced by decreased tissue histological damage scores and improved biochemical markers for liver and kidney function [42].

The compound demonstrates robust suppression of NF-κB activation, a central regulator of inflammatory gene expression. Biotransformed ginseng berry extract enriched with Rg2 showed significant suppression of NF-κB activation and inflammatory target genes in LPS-stimulated RAW 264.7 cells. This inhibition operates through prevention of IκBα phosphorylation and degradation, thereby blocking nuclear translocation and subsequent transcription of inflammation genes such as TNF-α, IL-1β, and IL-6. Immunofluorescence staining further validated this mechanism by demonstrating reduced NF-κB nuclear accumulation following Rg2 treatment [158].

Cardiovascular inflammation represents a major therapeutic target where Rg2 exhibits particular efficacy. In myocardial ischemia/reperfusion models, Rg2 treatment significantly inhibited the expression of TNFα, IL-1β, IL-6, and monocyte chemoattractant protein-1 (MCP-1). The cardioprotective mechanism involves precise regulation of TAK1 phosphorylation to block receptor-interacting protein (RIP)1/3 necrosome formation, ultimately reducing myocardial ischemia/reperfusion (MI/R)-induced necroptosis and associated inflammatory responses [155]. Additional cardioprotective pathways include Rg2’s attenuation of myocardial ischemia/reperfusion injury through reduction in oxidative stress and inflammation via SIRT1 signaling [159].

The immunomodulatory effects extend to regulation of cellular survival pathways. Rg2 significantly neutralized cardiomyocyte apoptosis induced by doxorubicin in a dose-dependent manner through the PI3K/AKT pathway. This protective effect correlates with upregulated protein kinase B phosphorylation and inhibited p53 expression, demonstrating Rg2’s ability to modulate both inflammatory and apoptotic responses [160]. In bone metabolism, RANKL-induced phosphorylation of MAPKs was decreased by ginsenoside Rg2 in bone marrow macrophages, indicating suppression of inflammatory osteoclastogenesis [31].

Hepatic inflammation and fibrosis represent additional therapeutic applications where Rg2 demonstrates significant efficacy. Rg2 significantly improved pathological changes in liver tissue induced by choline-deficient, amino acid-defined, high-fat diet, inhibiting serum transaminase, plasma lipopolysaccharide, and liver hydroxyproline levels. The antifibrotic effects are mediated through activation of the AKT/mTOR signaling pathway and inhibition of liver expression of autophagy-related proteins [156]. These findings—encompassing TLR4 antagonism, NF-κB suppression, tissue-specific protection, and metabolic regulation—collectively establish Rg2 as a multi-target anti-inflammatory agent capable of modulating diverse inflammatory pathways while promoting tissue protection and repair across multiple organ systems.

#### 3.5.4. Clinical Translation Potential and Safety Profile

The clinical translation of ginsenoside Rg2 faces inherent pharmacokinetic challenges common to natural product development, yet demonstrates significant potential through innovative solutions and comprehensive preclinical validation. Systematic metabolic studies have revealed that Rg2 undergoes extensive biotransformation through CYP450-mediated pathways with oxidative modifications occurring exclusively at the C17-side-chain while preserving structural integrity. Notably, this metabolic profile generates bioactive metabolites M1, M3, and M4 that function as SIRT1 activators with effects superior to the parent compound and comparable to resveratrol, indicating beneficial bioactivation rather than detoxification [161].

Addressing bioavailability limitations has been achieved through advanced formulation strategies. Lipid nanoparticle formulations containing ginsenoside Rg2 achieved superior mRNA encapsulation efficiency through hydrogen bonds formed between Rg2’s hydroxyl groups and mRNA’s polar functional groups [162]. These formulation advances represent a paradigm shift toward engineered delivery systems that optimize therapeutic potential while overcoming traditional pharmacokinetic barriers.

Comprehensive safety evaluation establishes the foundation for clinical development. Acute oral toxicity studies demonstrated no observable adverse effects at single doses up to 10 g/kg in mice, while subchronic evaluation through 28-day repeated dosing at 1.75, 3.5, and 5 g/kg/day revealed minimal toxicity with beneficial metabolic effects. Remarkably, total cholesterol levels decreased dramatically while prothrombin time increased, suggesting therapeutic benefits rather than adverse effects [32]. These findings indicate an exceptionally wide therapeutic window that supports clinical development.

Target indication selection is guided by robust preclinical efficacy data across multiple therapeutic areas. Cardiovascular applications represent the most advanced indication, with demonstrated cardioprotective effects against doxorubicin-induced cardiomyocyte apoptosis through PI3K/AKT pathway activation [160]. Hepatoprotective applications offer additional potential, with Rg2 demonstrating significant improvement in pathological changes through AKT/mTOR-mediated autophagy regulation [156]. Neurological applications represent an emerging frontier, as combined Rg1 and Rg2 studies demonstrate autophagy activation and oxidative stress attenuation in neuroblastoma cells overexpressing Aβ(1-42) [163].

The clinical development strategy is well-positioned based on favorable safety profiles demonstrated in preclinical studies, proven efficacy across multiple indications as discussed above, and innovative formulation solutions that address bioavailability challenges. Priority should be placed on cardiovascular indications given the strength of mechanistic data, with hepatic and neurological applications representing sequential development opportunities.

The immunomodulatory mechanisms and therapeutic applications of these five rare ginsenosides are summarized in Table 2.

## 4. Comparative Analysis and SAR

### 4.1. Standardized Indirect Comparison

Despite the absence of standardized head-to-head comparisons, careful analysis of available data (building on individual mechanisms described in Section 3) reveals a fundamental dichotomy among these five rare ginsenosides: F1 uniquely enhances immune responses while the other four consistently demonstrate various immunosuppressive mechanisms.

Ginsenoside F1 demonstrates distinctive immunostimulatory profile through comparative studies. At 10 μM concentration, F1 increased NK cell cytotoxicity by 87% against YAC-1 lymphoma cells, with the effect mediated through IGF-1 receptor-dependent mechanisms. NK cell depletion using anti-asialo-GM1 antibody completely abolished the anti-tumor effects of F1, confirming NK cell dependency [25]. In chronic rhinosinusitis models, F1 at 50 mg/kg intraperitoneally significantly attenuated eosinophilic inflammation, while dexamethasone at 2 mg/kg suppressed macrophage activation through different mechanisms [27].

In contrast, the remaining four ginsenosides—Rg5, Rk1, Rh1, and Rg2—all exhibit anti-inflammatory properties [42,64,66,124], albeit through distinct molecular targets and pathways. Ginsenoside Rg5’s anti-inflammatory effects operate through direct TLR4 antagonism [88]. In LPS-stimulated BV2 microglial cells, Rg5 significantly suppressed nitric oxide production and reduced proinflammatory TNF-α secretion in a dose-dependent manner [64]. When combined with Rk1 in HMGB1-mediated septic models, treatment at 20 mg/kg achieved 70% survival rate compared to 20% in controls [66]. Additionally, Rg5 specifically activated IGF-1 receptor with an EC50 value of approximately 90 nM, promoting angiogenesis and vasorelaxation [65].

Ginsenoside Rk1 demonstrated the broadest anti-inflammatory coverage through its multi-pathway modulation capabilities. In HMGB1-mediated septic responses, Rk1 at 20 mg/kg significantly reduced TNF-α and IL-6 production through inhibition of NF-κB and ERK1/2 activation [66]. The compound’s anti-thrombotic activity was evident as 10 μM Rk1 decreased thromboxane B2 levels by 77% in washed platelets, showing stronger anti-platelet effects than aspirin at 100 μM [113].

Ginsenoside Rh1 specialized in anti-allergic responses with remarkable potency. In passive cutaneous anaphylaxis models, Rh1 at 25 mg/kg achieved 87% inhibition, significantly exceeding disodium cromoglycate’s 31% inhibition at the same dose [124]. The compound’s metabolite, PPT, further contributed to efficacy by suppressing TNBS-induced colitis in mice at 20 mg/kg dose. In ovalbumin-induced asthmatic mice, Rh1 treatment at 100 mg/kg comprehensively modulated the immune response by significantly suppressing elevated levels of Th2 cytokines including IL-4, IL-5, and IL-13 [138].

Ginsenoside Rg2, particularly when combined with Rh1, demonstrated potent anti-inflammatory synergy. The combination synergistically attenuated LPS-induced inflammation through selective inhibition of p38 MAPK and STAT1 activation, while simultaneously blocking PKCδ translocation to the plasma membrane and reducing inflammatory cytokine production [42]. Beyond inflammation, Rg2 demonstrated cardioprotective effects against trastuzumab-induced toxicity by inducing autophagy and inhibited osteoclastogenesis by downregulating NFATc1, c-Fos, and MAPK pathways [107].

Notably, synergistic effects emerged when combining these immunosuppressive ginsenosides. The Rg5 and Rk1 combination achieved superior sepsis survival rates in co-administration studies [66], while Rg2 and Rh1 demonstrated enhanced organ protection through complementary pathway inhibition [42]. These synergies suggest potential for rational combination strategies in therapeutic applications.

The structural basis for this functional dichotomy relates directly to specific glycosylation patterns. F1’s sugar moiety at C-20 is required for NK cell activation, whereas that at C-6 is dispensable. This C-20 glucose configuration appears to function as a molecular switch: its presence in F1 confers immunostimulatory activity, while its absence or different positioning in other ginsenosides correlates with anti-inflammatory properties. This SAR provides crucial insights for understanding why F1 alone among these rare ginsenosides enhances rather than suppresses immune responses [25].

The absence of standardized protocols for direct comparison remains a significant limitation in definitively ranking these compounds’ relative potencies [107]. Future studies employing uniform experimental conditions across all five ginsenosides would provide more definitive comparative data and enable precise therapeutic optimization.

### 4.2. Mechanistic Pathway Mapping

The molecular mechanisms underlying the immunomodulatory effects of these five rare ginsenosides reveal a hierarchical organization of pathway targeting, from upstream receptor interactions to downstream transcriptional regulation. This mechanistic diversity explains their distinct biological activities and provides insights into their therapeutic applications.

At the upstream receptor level, ginsenosides demonstrate fundamentally different targeting strategies. Ginsenoside F1 operates through a unique enhancement mechanism, promoting cytotoxic activity of NK cells via IGF-1-dependent mechanism. IGF-1 blockade antagonized NK cell potentiation by F1, while IGF-1 treatment alone recapitulated the effect. F1-treated NK cells showed enhanced phosphorylation of AKT rather than ERK, induced by co-engagement of NKG2D and 2B4 receptors at 10 μM concentration, with the sugar moiety attached at C-20 being required for NK cell activation whereas that at C-6 is dispensable [25]. This IGF-1R-mediated enhancement pathway represents the only immune-stimulatory mechanism among the five compounds.

In contrast, the remaining four ginsenosides target inflammatory pathways through direct TLR4 receptor interactions. Ginsenoside Rg5 ameliorates lung inflammation in mice by inhibiting the binding of LPS to TLR4 on macrophages [88]. Molecular docking studies demonstrate ginsenoside Rb3 attenuates LPS-mediated inflammation through direct inhibition of TLR4 signaling pathway, with binding energy of −8.79 kcal/mol to the TLR4/myeloid differentiation factor 2 (MD2) complex [75]. Rare ginsenosides Rg2 and Rh1 synergistically block LPS-mediated PKCδ translocation to the plasma membrane, resulting in p38-STAT1 activation and NF-κB translocation, while inhibiting the binding of LPS to TLR4 on peritoneal macrophages and suppressing the TLR4-mediated signaling pathway [42]. This direct TLR4 targeting creates a convergent suppressive mechanism shared by multiple ginsenosides.

At the intermediate signaling level, all compounds modulate NF-κB and MAPK pathways but through distinct molecular approaches. F1 demonstrates a unique mechanism by inducing A20-mediated suppression of NF-κB signaling, significantly inhibiting G-CSF, ICAM-1, MIP-1δ, IL-1α, IL-15, and IL-16 levels [82]. Heat-processed ginseng containing Rg3, Rg5, and Rk1 increased macrophage activation which was regulated by the ERK/c-Jun pathway in RAW264.7 cells, with the ERK inhibitor completely suppressing the effect on IL-6 and TNF-α production [164]. Ginsenoside Rg5 suppresses the DNA binding activities of both NF-κB and AP-1, which are key transcription factors controlling inflammatory reactions [64]. RANKL-induced phosphorylation of mitogen-activated protein kinases was decreased by ginsenoside Rg2 in bone marrow macrophages [31]. These differential MAPK and NF-κB modulation patterns create compound-specific signaling signatures.

At the downstream transcriptional level, ginsenosides Rk1 and Rg5 demonstrate coordinated effects through SIRT1-mediated pathways, suppressing HMGB1-mediated septic responses through SIRT1-mediated deacetylation of HMGB1 and inhibition of TNF-α and IL-6 production as well as NF-κB and ERK 1/2 activation, with their combination showing enhanced suppression of HMGB1-mediated inflammatory responses compared to individual compounds [66]. Multiple ginsenosides, including ginsenoside Rg5, can enhance mitochondrial biogenesis and mtDNA generation in multiple types of normal tissues by activating the Sirt1/PGC-1α signaling pathway [165]. This SIRT1-mediated regulation represents a convergent downstream mechanism for metabolic and inflammatory control.

The mechanistic analysis reveals both convergent and divergent pathway utilization patterns. LPS binding-induced TLR4 activation leads to the activation of various intracellular signaling pathways such as MAPK and JAK-STAT signaling pathways, which play an important role in inflammatory responses [42]. While F1’s IGF-1R-mediated enhancement pathway creates a unique immunostimulatory profile, the other four compounds converge on TLR4 suppression but diverge in their intermediate signaling and downstream transcriptional targets. Ginsenoside Rh1 possesses antiallergic and anti-inflammatory activities [124], representing specialized pathway targeting for allergic responses. This hierarchical pathway organization explains why F1 uniquely enhances immune responses while others provide targeted suppression, establishing the molecular foundation for their distinct therapeutic applications and synergistic potential.

### 4.3. Structural and Mechanistic Basis for F1’s Unique Immunostimulatory Activity

F1’s unique enhancement properties stem from its ability to activate the IGF-1/IGF1R pathway, which fundamentally promotes cellular activation and proliferation. The structural basis for this distinction lies in the specific sugar configuration: the sugar moiety attached at the C-20 is required, whereas that at the C-6 is dispensable or inhibitory, for promoting NK cell effector functions. This C-20 glucose configuration enables F1 to trigger IGF-1 signaling, as evidenced by the fact that NK cell potentiation by F1 was antagonized by IGF-1 blockade and recapitulated by IGF-1 treatment, confirming the involvement of IGF-1 [25].

The IGF-1 pathway inherently drives cellular activation rather than suppression. IGF-1 is a crucial mitogenic factor with important functions that activates a complex signaling network promoting cell proliferation, epithelial to mesenchymal transition (EMT) and inhibits apoptosis [166]. In the context of immune enhancement, F1 activates the IGF-1/IGF1R pathway to promote angiogenesis, which reduces cerebral ischemia [167]. This pathway activation directly correlates with enhanced immune function, as demonstrated when peanut sprout extracts cultivated with fermented sawdust medium (PSEFS) upregulated lytic enzymes and cytokines, such as GZMBv and IFNγ, respectively, in NK cells by activating the MAPK pathway. Among these receptors, NKp30 and NKp46 are known to play a crucial role in NK-mediated cytolytic activity through CD3ζ signaling in activated NK cells [76].

In stark contrast, other rare ginsenosides consistently activate anti-inflammatory pathways that suppress immune responses. The inhibitory effect of ginsenosides from steamed ginseng-leaves and flowers on the LPS-stimulated IL-12 production reveals the magnitude of this suppression: ginsenoside Rg6 and ginsenoside F4 exhibited particularly potent inhibitory effect on LPS-induced IL-12 production with the inhibition values of 80 and 82%. This represents near-complete immune suppression, with other ginsenosides ST1, SL2, SL3, Rh3, Rk2, and Rs4 showing moderate suppressive effects with inhibition rates ranging from 63–73% [168].

The mechanistic basis for this suppression involves NF-κB and AMPK pathway modulation, which fundamentally oppose IGF-1 signaling. Rh1 affects the levels of iNOS and COX-2 protein and suppresses IFN-γ and NF-κB mediated JAK/STAT and ERK activation. In parallel, ginsenoside Rg3 and Rg1 instigate the AMPK pathway, elevating the levels of phosphoacetyl-CoA carboxylase (p-ACC) and p-AMPK [169]. By modulating energy metabolism and inflammatory responses, AMPK exerts significant influence on tumor cell development, while also playing a pivotal role in tumor immunotherapy by regulating immune cell activity and function [170].

The critical molecular switch lies in sugar positioning and number. The biological activities of ginsenosides are related to their structures, especially to the type of aglycone and the number of sugars linked to the core structure [171]. Enhanced anticancer activity emerges when sugar units are found in the C-3 or C-20 position compared to sugar units at the C-6 position [172]. This positional specificity explains why F1, with its optimal C-20 glucose, selectively activates IGF-1 pathways while other configurations favor suppressive pathways. The stereochemistry further fine-tunes pathway selection, as the natural product form in *Panax ginseng* is believed to be the 20(S)-diastereomer, which exhibits higher bioactivity than the 20(R)-diastereomer. Only the 20(S)-diastereomers of Rg3 and Rh2 are active as positive allosteric modulators (PAMs) at human P2X purinoceptor 7 (hP2X7) [173].

The number of sugar moieties acts as a critical determinant of enhancement versus suppression. As the number of sugar moieties increases, the cytotoxic and anti-cancer activity of the ginsenoside decreases. Ginsenosides with four or more sugar molecules, such as Rb1 and Rc, show no significant anti-proliferative effects [174]. The hydroxyl group at the C-20 location with a single sugar moiety on the ginsenoside molecule plays a dynamic role in inhibiting RLAR and reduces the action of two or more sugar moieties [175]. Notably, the effect of F1 on NK cell effector functions was more potent than that of Rg1 [167], demonstrating F1’s superiority even among enhancement-capable ginsenosides.

While the precise mechanism of NK cell potentiation by ginsenosides requires further investigation [25], the opposing outcomes are clear: F1’s potent enhancement contrasts sharply with 80–82% suppression by other ginsenosides, revealing fundamentally different biological programs activated by subtle structural differences. This structure–function relationship demonstrates that F1’s C-20 glucose positioning acts as a molecular switch directing compounds toward either IGF-1-mediated cellular activation or NF-κB/AMPK-mediated immune suppression, explaining why F1 uniquely enhances immune function while structurally similar compounds consistently suppress it.

The molecular basis for F1’s selective IGF-1R activation over TLR4 antagonism warrants further mechanistic consideration. The single glucose moiety at C-20 position in F1, without additional glycosylation at C-6, creates a unique molecular topology that may facilitate selective interaction with growth factor receptors rather than pattern recognition receptors. IGF-1R, a transmembrane tyrosine kinase receptor, recognizes specific glycosylation patterns that modulate cellular proliferation and survival [176]. The C-20 monoglucosylation in F1 may structurally mimic endogenous growth factor ligands, as glycosylation plays a critical role in growth factor-receptor interactions [177]. This structural mimicry hypothesis aligns with the evidence presented above that F1-induced NK cell activation is specifically mediated through IGF-1R signaling [25]. The absence of C-6 glycosylation in F1 likely prevents steric hindrance that would otherwise occlude the IGF-1R ligand-binding domain, while the single C-20 glucose provides the necessary molecular recognition element for receptor activation. This differential receptor targeting based on precise glycosylation patterns represents a compelling example of how subtle structural variations in natural products can fundamentally alter their immunological outcomes, shifting from suppression to stimulation through distinct receptor pathways.

## 5. Cancer Immunotherapy Applications

### 5.1. F1: Multitarget Anticancer Mechanisms

Ginsenoside F1 demonstrates anticancer, anti-aging, and antioxidant properties with proven ability to activate NK cells effectively (mechanism detailed in Section 3.1.2) [63]. This compound represents a promising therapeutic agent whose mechanisms address both direct cellular effects and immune system enhancement. Unlike conventional single-target therapies, F1 exhibits a unique profile combining direct cytotoxic effects with immune surveillance amplification, positioning it as a potential candidate for comprehensive cancer treatment strategies.

The direct anticancer activity of F1 manifests through selective cytotoxicity against malignant cells while sparing normal tissues. In B16 melanoma cells, F1 suppressed proliferation by 60% at 200 μg/mL while reducing normal HEK293 cell viability by only 30%, indicating differential sensitivity that warrants further validation across diverse cancer cell lines. This selectivity extends to the molecular level, where F1 induced morphological alterations and cellular clustering in melanoma cells through biphasic modulation of proliferation-related signaling pathways—initially activated at 1 h but subsequently downregulated at 12 h [178].

Complementing its direct effects, F1 potently enhances immune-mediated tumor Complementing its direct effects, F1 potently enhances immune-mediated tumor elimination through selective activation of natural killer cells. Among 15 ginsenosides evaluated, F1 showed the most robust enhancement of NK cell cytotoxicity against diverse cancer cell types via an IGF-1 dependent mechanism [25]. The clinical relevance of this immune enhancement was established through NK cell depletion studies, where removal of NK cells completely abolished F1’s therapeutic efficacy in disease models, confirming NK cell activation as essential for F1’s anticancer activity [27].

F1’s immunomodulatory effects extend beyond NK cell activation to encompass broader immune coordination. The compound enhanced IL-13 production from epidermal γδ T cells, which subsequently suppressed melanogenesis-related enzymes including tyrosinase and dopachrome tautomerase [84]. This multi-immune cell coordination suggests that F1 may modulate diverse immune responses beyond NK cell activation, although the clinical significance of these interactions requires further investigation.

A critical advantage of F1 lies in its cytoprotective properties that may enhance the tolerability and efficacy of combination cancer therapies. F1 protected normal keratinocytes from UV-induced apoptosis by maintaining anti-apoptotic protein levels of Bcl-2 and brain-specific homeobox/POU domain protein 3a (Brn-3a) [179]. More significantly for cancer treatment applications, F1 substantially reduced drug-induced hepatotoxicity through activation of the Keap1/Nrf2/ARE antioxidant pathway, suggesting potential to mitigate chemotherapy-related organ damage without compromising therapeutic efficacy [87].

The therapeutic potential of F1’s immune-enhancing properties gains additional significance from clinical evidence demonstrating the consequences of compromised immune surveillance. Studies show that environmental factors like cigarette smoke can impair NK cell-dependent tumor surveillance, leading to increased tumor burden and highlighting the clinical need for immune-restoring interventions [180]. F1’s selective enhancement of NK cell function without suppressing other immune responses distinguishes it as a potential adjuvant for restoring effective cancer immunosurveillance in compromised patients [27].

### 5.2. Rk1’s Multifaceted Anticancer Mechanisms

Ginsenoside Rk1 demonstrates significant capacity to modulate the tumor microenvironment through targeted effects on immune cell populations and multiple signaling pathways. The tumor microenvironment consists of vasculature, extracellular matrix, cytokines and growth factors, and many different populations of stromal cells, such as myeloid-derived suppressor cells and tumor-associated macrophages [181].

Rk1’s primary anticancer mechanism involves immune checkpoint modulation that enhances anti-tumor immunity. The compound effectively reduces programmed death-ligand 1 (PD-L1) expression in non-small cell lung cancer by inhibiting the NF-κB pathway. This checkpoint inhibition represents a critical mechanism for overcoming tumor immune evasion, as PD-L1 upregulation is a primary method by which tumors evade immune surveillance. The compound’s ability to target this pathway positions it as a promising adjuvant for cancer immunotherapy [182].

Advanced drug delivery systems have transformed Rk1’s therapeutic potential through enhanced tumor targeting. Rk1-modified liposomes achieved 97.24 ± 0.75% encapsulation efficiency with superior tumor accumulation. The enhanced tumor-targeting ability stems from exposed Rk1 glycosides that reduce plasma protein adhesion, prolonging circulation time, while simultaneously binding to GLUT1 protein highly expressed in tumor cells, thereby promoting accumulation in tumor tissues. These formulations achieved significant reduction in average tumor volume compared to conventional cholesterol-containing liposomes [117].

Systemic immunomodulation by Rk1 restores anti-tumor immune balance. Heat-processed American ginseng saponins (AGS-H) treatment containing rare ginsenosides including Rk1 significantly increased spleen index and white blood cell count, with concurrent upregulation of IL-2 and IL-10 immune cytokines. The treatment restored immune homeostasis by significantly improving the CD4+/CD8+ ratio while significantly inhibiting CD4+CD25+ levels, indicating improved immune balance. CD4+ cells are markers on helper T cells that enhance humoral and cellular immunity, while CD8+ T cells specifically kill infected and dysfunctional cells [183].

The compound’s anticancer effects operate through multiple complementary mechanisms. Cancer cells can be killed and cancer growth can be suppressed by Rk1, which exhibits potent effects on promoting apoptosis of cancer cells including liver cancer, lung cancer and gastric cancer [63]. Beyond direct anti-tumor activity, Rk1 shows significant effects on anti-septicaemia, addressing a critical complication that commonly occurs in immunocompromised cancer patients [183]. This dual functionality makes Rk1 particularly valuable for cancer patients who often suffer from concurrent infections due to immunosuppression, as demonstrated in prostate cancer models where Rk1 treatment effectively relieved concurrent Candida albicans infection burden while maintaining anti-tumor efficacy [117].

### 5.3. Strategic Combination Approaches for Ginsenoside-Based Cancer Therapy

The development of strategic combination approaches represents a critical advancement in optimizing ginsenoside-based cancer immunotherapy. These approaches leverage the multitarget properties of ginsenosides to address the complex challenges of cancer treatment through synergistic mechanisms. Ginsenoside F1 enhances NK cell cytotoxicity through IGF-1-dependent mechanisms, promoting cancer surveillance in mouse models of lymphoma clearance and metastatic melanoma [25]. In parallel, ginsenoside Rg5 promotes breast cancer cell apoptosis by inducing G0/G1 cell cycle arrest in MCF-7 and MDA-MB-453 human breast cancer cell lines. The synergistic potential of natural compounds has been validated through studies showing that combined biochanin A, quercetin, and epigallocatechin-3-gallate (EGCG) at 5 mg/kg led to improved efficacy comparable to that of 15 mg/kg biochanin A alone [184], supporting the principle of synergistic natural product interactions.

Tumor microenvironment normalization represents another strategic approach where ginsenosides can enhance immunotherapy efficacy. Ginsenoside Rh4 effectively inhibits PD-L1 expression by regulating histone deacetylase 2 (HDAC2)-mediated JAK/STAT signaling pathways in breast cancer cells, while demonstrating high binding affinity with HDAC2 through molecular docking analysis [185]. The surface chemistry of Rg5-based delivery systems further contributes to efficacy: due to the disaccharide sugar moiety attached to the C-3 position of Rg5, the surface of the prepared polymer micelle drug delivery system is covered by hydrophilic sugar moieties, enhancing hydrophilicity and prolonging the residence time of the formulation in the circulatory system [186]. This enhanced circulation can be combined with vascular normalization strategies, as tumor microenvironment (TME) normalization improves immunotherapy by modulating tumor microenvironment via cytotoxic T cells [187].

Low-dose chemotherapy combinations with ginsenosides offer significant advantages in reducing systemic toxicity while maintaining therapeutic efficacy. Constant effort has been made toward achieving better therapeutic benefits of paclitaxel antitumor effects in cancer by reducing its dose in combination with other chemotherapeutic agents [188]. In this context, ginsenoside Rg3 inhibits NF-κB signaling in triple-negative breast cancer through both in vivo and in vitro studies [189]. The synergistic effects extend to paclitaxel resistance, where ginsenosides combined with paclitaxel exert enhanced antitumor activity [186], while ginsenoside Rg5 sensitizes paclitaxel-resistant human cervical adenocarcinoma cells to paclitaxel and enhances the anticancer effect of paclitaxel [190].

Sequential therapy protocols utilizing ginsenosides can optimize treatment outcomes by addressing different phases of cancer progression. Ginsenoside Rh4 modulates gut microbiota-mediated bile acid metabolism to inhibit colorectal cancer development [191], suggesting potential for maintenance therapy approaches. Multi-target approaches combining ginsenosides with conventional therapies enhance therapeutic efficacy, as demonstrated by the development of ginsenoside-based multifunctional liposomal delivery systems for combination therapy [192]. These integrated strategies—encompassing immune enhancement, TME normalization, chemotherapy sensitization, and sequential protocols—establish a comprehensive framework for ginsenoside-based cancer therapy that addresses both primary tumors and systemic disease.

**Figure 3:**
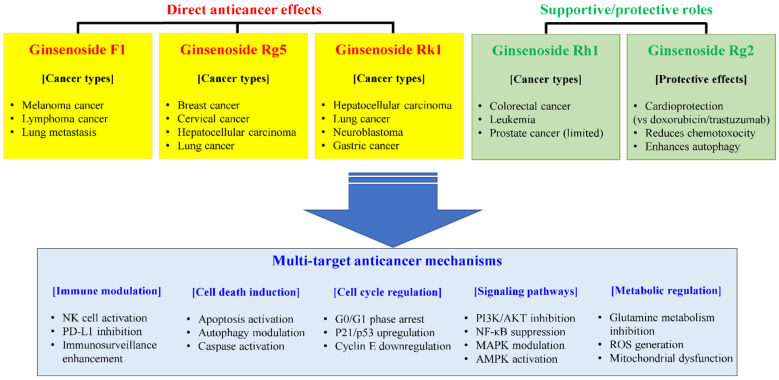
Cancer types and multi-target anticancer mechanisms of rare ginsenosides. Direct anticancer effects are demonstrated by F1, Rg5, and Rk1. Rh1 and Rg2 provide primarily supportive/protective roles in cancer therapy. The central panel shows multi-target mechanisms including immune modulation, cell death induction, cell cycle regulation, signaling pathway modulation, and metabolic reprogramming. NK, natural killer; PD-L1, programmed death-ligand 1; PI3K, phosphatidylinositol 3-kinase; AKT, protein kinase B; NF-κB, nuclear factor-kappa B; MAPK, mitogen-activated protein kinase; AMPK, adenosine monophosphate-activated protein kinase; ROS, reactive oxygen species.

To provide a comprehensive overview of the anticancer activities of these rare ginsenosides, we have summarized their effects on different cancer types and their multi-target mechanisms (Figure 3). The analysis reveals a clear distinction between compounds with direct anticancer effects (F1, Rg5, and Rk1) and those serving primarily supportive roles (Rh1 and Rg2). F1 demonstrates unique immunomodulatory properties through NK cell activation via IGF-1-dependent mechanisms [25], while Rg5 and Rk1 exhibit broad-spectrum cytotoxicity across multiple cancer types including breast cancer, cervical cancer, and hepatocellular carcinoma [114,193,194]. The multi-target nature of these compounds, affecting immune modulation [25], cell death pathways [195], cell cycle regulation [193], signaling cascades including PI3K/AKT and NF-κB inhibition [92], and metabolic processes such as glutamine metabolism [111], represents a significant advantage over conventional single-target therapies. Notably, Rk1 demonstrates PD-L1 inhibition in lung cancer cells [196], suggesting its potential in cancer immunotherapy. The supportive roles of Rh1 and Rg2 are equally important, with Rh1 enhancing dexamethasone’s anti-inflammatory effects [130] and Rg2 providing cardioprotection against chemotherapy-induced toxicity from doxorubicin and trastuzumab [160,197]. These multi-faceted anticancer mechanisms, combined with the observed synergistic effects such as the 70% survival rate with Rg5/Rk1 combination in sepsis models [107], position rare ginsenosides as promising candidates for overcoming drug resistance in modern cancer therapy.

These multi-faceted anticancer mechanisms, combined with their favorable safety profiles and ability to enhance conventional chemotherapy while reducing its toxicity, position rare ginsenosides as promising candidates for integration into modern cancer therapeutic strategies.

## 6. Applications in Inflammatory and Autoimmune Diseases

### 6.1. Allergic Diseases—Rh1 Dominance

#### 6.1.1. Mechanisms of Anti-Allergic Action

Ginsenoside Rh1 emerges as the most potent anti-allergic specialist among rare ginsenosides (as established in Section 3.4), functioning in direct contrast to F1’s unique immune enhancement by providing comprehensive allergic suppression through mast cell stabilization and inflammatory pathway inhibition. Metabolite ginsenosides including Rh1 suppress allergic reactions such as passive cutaneous anaphylaxis, scratching, and asthma more potently than their parental ginsenosides Rb1, Rg3, and Re [134].

At the molecular level, Rh1’s anti-allergic efficacy stems from targeted suppression of key inflammatory transcription factors and enzymes. Ginsenoside Rh1 inhibited inducible nitric oxide synthase and COX-2 protein expression in RAW264.7 cells while simultaneously suppressing activation of the transcription factor NF-κB in nuclear fractions [124]. The compound also activates protective pathways, as Rh1 upregulated phase II antioxidant enzymes including heme oxygenase-1 through MAPK pathways and Nrf2/ARE signaling in rat primary astrocytes [132].

These molecular effects translate into potent mast cell stabilization, a cornerstone of anti-allergic therapy. Ginsenoside Rh1 achieved 87% inhibition of IgE-mediated passive cutaneous anaphylaxis reaction at 25 mg/kg, significantly exceeding the 31% inhibition produced by disodium cromoglycate at the same dose, with membrane-stabilizing properties directly confirmed by differential scanning calorimetry [124].

This superior efficacy extends to chronic allergic conditions, where fermented red ginseng containing Rh1 at 200 mg/kg significantly inhibited scratching frequency by 45% in the scratching behavioral experiment, while also inhibiting degranulation and reducing TNF-α and IL-4 expression in RBL-2H3 cells [135]. In inflammatory bowel models, ginsenoside Rh1 suppressed IL-1β and TNF-α expression in TNBS-induced colitis mice [126].

#### 6.1.2. Disease Model Efficacy

Ginsenoside Rh1 demonstrates therapeutic efficacy across multiple allergic disease models through suppression of inflammatory responses and modulation of immune pathways [128,138].

In ovalbumin-induced asthma models, Rh1 treatment comprehensively ameliorates both physiological and immunological parameters. Ginsenoside Rh1 significantly alleviated lung resistance and airway resistance while reducing the number of total inflammation cells, eosinophils, neutrophils, and lymphocytes in bronchoalveolar lavage fluid of asthmatic mice. The therapeutic effects extended to structural improvements, with reduced airway remodeling and collagen deposition in lung tissues. At the molecular level, Rh1 restored immune balance by reversing the increase in eotaxin, IL-4, IL-5, IL-13, and IL-33 and the decrease in IL-12 and IFN-γ in both bronchoalveolar lavage fluid and serum of ovalbumin-exposed mice [138].

The anti-asthmatic mechanisms involve multiple signaling pathway interventions. Rh1 inhibits immune cell infiltration and prevents allergic asthma by blocking the activation of the MAPK, AKT, and NF-κB pathways [198]. This multi-pathway inhibition explains why ginsenosides including Rh1 suppress allergic reactions such as asthma more potently than parental ginsenosides, with metabolites showing enhanced therapeutic activity compared to their precursors [134].

In allergic rhinitis models, Rh1 effectively targets both tissue pathology and underlying immune dysfunction. The compound alleviates house dust mite-induced nasal mucosal epithelial thickening and eosinophil infiltration by modulating mitochondrial autophagy via the AMPK/ULK1/FUNDC1 signaling pathway. Flow cytometry analysis revealed that Rh1 significantly curbed the rise in IL-4+ CD4+ cells in allergic rhinitis mice while elevating IFN-γ+ CD4+ cell proportions, demonstrating restoration of Th1/Th2 immune balance [128]. Clinical relevance was supported by findings that eosinophil counts in nasal smears were significantly reduced in the Korean red ginseng treatment group, validating the anti-allergic efficacy of ginseng-derived compounds [199].

Collectively, these findings suggest that Rh1 may serve as a broad-spectrum anti-allergic agent, though further clinical validation is needed.

### 6.2. Inflammatory Disease—Rk1’s Intestinal Focus

#### 6.2.1. Anti-Inflammatory Mechanisms

Ginsenoside Rk1 exerts anti-inflammatory effects through direct inhibition of NF-κB, a central regulator of inflammatory responses. Rk1 has an anti-inflammatory effect by inhibiting NF-κB levels in vitro models, as assessed using luciferase assay [107]. This inhibitory mechanism operates through specific molecular targeting, as ginsenoside Rk1 inhibits NF-κB by targeting Annexin A2, following the same mechanism identified for Compound K and Rg5 [114].

The anti-inflammatory activity extends to comprehensive pathway modulation involving PI3K/AKT signaling cascades. Rk1 suppresses the PI3K/AKT/NF-κB signaling pathways in vitro and in vivo, with the PI3K, AKT, IκBα, and NF-κB phosphorylation levels being notably enhanced in UVB-irradiated human keratinocyte (HaCaT) cells before treatment [110]. Beyond NF-κB regulation, Rk1 inhibited the effects of collagen, arachidonic acid (AA), and U46619-induced platelet aggregation, indicating broader anti-inflammatory applications [107].

Oxidative stress reduction contributes significantly to Rk1’s anti-inflammatory mechanisms by breaking the inflammation-oxidation cycle. Rk1 administration significantly attenuated oxidative stress by suppressing ROS overproduction and strengthening the activities of antioxidant enzymes [110]. In black ginseng-derived preparations, ginsenoside Rk1 (purity > 95%) showed protective effects, with GSH levels elevated and MDA significantly decreased after Rk1 treatment [22].

Multiple signaling pathway targeting provides the mechanistic basis for Rk1’s comprehensive anti-inflammatory profile. Rk1 and Rg5 shared 44 putative targets associated with hepatocellular carcinoma, with enrichment analysis revealing that Rk1 may induce effects through inhibition of MAPK and NF-κB signal pathways [114]. In epithelial–mesenchymal transition models, ginsenosides Rk1 and Rg5 inhibit transforming growth factor-β1-induced transition through modulation of Smad family proteins (Smad), NF-κB, ERK1/2, p38 MAPK, and JNK signaling pathways [108].

Clinical translation potential is supported by systematic evidence across multiple experimental models. A systematic review of 28 studies found that 21 studies were related to the effectiveness of Rk1 only and seven studies reported on combined effects of Rk1 and Rg5, with the most common study design being in vitro studies [107]. The consistent anti-inflammatory effects across diverse experimental systems support Rk1’s therapeutic potential for inflammatory conditions requiring multi-pathway intervention. These anti-inflammatory mechanisms provide the foundation for subsequent barrier function restoration by reducing inflammatory damage to intestinal epithelium.

#### 6.2.2. Barrier Function Restoration

Building upon the anti-inflammatory effects described above, ginsenoside Rk1 demonstrates potential in intestinal barrier restoration, though direct mechanistic studies remain limited. Fermented cultured wild ginseng containing increased levels of rare ginsenosides including Rk1 improved intestinal barrier functions, recovery, permeability, and expression of tight junction protein genes in Caco-2 cells [200].

Rk1’s barrier protective capacity operates through stabilization of intercellular junctions. Rk1 exhibits anti-permeability activity through enhanced stability of tight-junction proteins at the boundary between cells [201]. This mechanism suggests that Rk1 contributes to maintaining epithelial barrier integrity by strengthening intercellular connections that regulate paracellular transport and maintain tissue compartmentalization.

The barrier protective effects of Rk1 extend beyond intestinal tissues, demonstrating broad applicability across different epithelial and endothelial systems. In vascular permeability studies, Rk1 ginsenoside reduced leakage of retinal vessels in diabetic mice and inhibited endothelial permeability caused by VEGF and other vasoactive factors such as thrombin and histamine in human retinal microvascular endothelial cells [202]. These findings demonstrate Rk1’s broad barrier-protective effects across different epithelial and endothelial systems, supporting its therapeutic potential for intestinal barrier dysfunction.

### 6.3. Sepsis—Individual Mechanisms

Sepsis progression involves a biphasic inflammatory response, with early mediators like TNF-α followed by late mediators including HMGB1, which provides a crucial therapeutic window for intervention [203]. Individual rare ginsenosides Rg5 and Rk1 demonstrate distinct mechanistic approaches to sepsis control through unique pathways.

Ginsenoside Rg5 provides upstream intervention by directly targeting the initial recognition phase of sepsis. Ginsenoside Rg5 ameliorates lung inflammation in mice by inhibiting the binding of LPS to TLR4 on macrophages [88]. In LPS-stimulated BV2 microglial cells, Rg5 significantly suppressed nitric oxide production and reduced proinflammatory TNF-α secretion in a dose-dependent manner, while mechanistically inhibiting the phosphorylation of PI3K/AKT and all three MAPKs (ERK, JNK, and p38) and suppressing the DNA binding activities of both NF-κB and AP-1 transcription factors [64].

While Rg5 targets upstream inflammatory initiation, ginsenoside Rk1 addresses downstream protective mechanisms critical for sepsis complications. Rk1 strongly inhibited permeability induced by VEGF and significantly reduced vessel leakiness through enhanced tight junction protein stability [112]. The compound also protects against oxidative injury via regulation of the PI3K/AKT/Nrf2/HO-1 pathway, as demonstrated in human melanocytes exposed to H_2_O_2_-induced oxidative stress [115]. These complementary mechanisms—Rg5’s receptor-level blockade and Rk1’s tissue protection—provide distinct therapeutic approaches for sepsis management. The combination of upstream inflammatory prevention and downstream organ protection suggests potential for rational combination strategies in sepsis treatment, where timing and mechanism of intervention critically determine outcomes.

To facilitate cross-comparison of in vivo efficacy across different ginsenosides and disease models, we compiled the key preclinical findings in Table 3. This summary demonstrates the consistent therapeutic potency of rare ginsenosides across diverse animal models, with effective doses ranging from 0.031 mg/kg for intravenous administration to 50 mg/kg for oral delivery.

### 6.4. Synergistic Effects and Combination Strategies

All synergistic effects discussed in this section were empirically demonstrated through direct co-administration of compounds in the cited studies, with statistical validation of greater-than-additive effects. Autoimmune diseases are characterized by complex inflammatory processes where inflammatory mediators play important roles in pathogenesis, making targeting of inflammatory mediators and pathways one of the emerging treatment strategies [204]. The traditional use of whole ginseng preparations, rather than isolated individual ginsenosides, provides scientific rationale for combination strategies. Evidence demonstrates that whole ginseng extract gives better protection against radiation-induced DNA damage than isolated ginsenoside fractions, supporting the concept that multiple bioactive compounds working together produce superior therapeutic effects [205]. Identifying effective and potent immunostimulants or immunosuppressants is critical for immune system modulation [206].

The individual anti-inflammatory profiles of emerging rare ginsenosides described in previous sections create opportunities for rational combination design Among these combinations, the pairing of Rg5 and Rk1 has emerged as a prototype for understanding synergistic mechanisms in neuroinflammation. Research demonstrates that the Rg5/Rk1 mixture produces enhanced effects compared to individual compounds in multiple inflammatory pathways. In neuroinflammatory models, this combination significantly ameliorated sleep disturbances via regulating GABAergic and serotoninergic signaling pathways, with Rg5 and Rk1 augmenting the GABA/glutamate (Glu) ratio and upregulating GABAA and GABAB receptor expression [99]. This dual neurotransmitter modulation demonstrates the potential of combination approaches in neuroinflammation.

The mechanistic rationale for Rg5/Rk1 synergy builds on their complementary potency profiles and distinct molecular targets. Ginsenoside Rk1 exhibits approximately two times higher anticancer activity than Rg5 when tested individually, yet their combination produces effects exceeding the sum of individual activities [107]. In N-methyl-D-aspartate (NMDA) receptor modulation studies, cotreatment with ginsenosides Rg3 and Rk1 proved more effective in receptor inhibition compared to either compound alone, suggesting different binding sites and additive mechanisms [207]. This differential targeting pattern indicates that emerging rare ginsenosides can achieve comprehensive pathway modulation while potentially reducing the doses required for therapeutic effect.

Practical implementation of combination strategies benefits from processing technologies that generate multiple rare ginsenosides simultaneously, creating naturally synergistic formulations. Ultrasonication-processed red ginseng extracts optimized at 100 °C for 12 h achieved the highest concentrations of ginsenosides Rg3 (0.803%), Rg5 (0.167%), and Rk1 (0.175%), creating inherently synergistic preparations [207]. Heat processing with specific amino acids further enhances rare ginsenoside generation, with the content of 20(S)-Rg3 in ginseng extract increasing to more than 30% when processed with valine, arginine, and alanine compared to normal heat processing [208]. These processing advances enable the development of standardized multi-component preparations for inflammatory disease treatment.

The broader implications of combination strategies extend beyond simple additive effects to include functional complementarity across different inflammatory pathways. For clinical translation, bioconversion technology for ginseng products containing preconverted metabolites including Rg3, Rk1, Rg5, and F2 is considered useful for avoiding individual variations in ginsenoside metabolism and promoting absorption [209]. This standardization approach supports the development of consistent therapeutic formulations for inflammatory and autoimmune disease management.

Beyond the demonstrated pairwise synergies, the concept of ‘entourage effect’—where multiple ginsenosides work together to produce effects greater than their individual contributions—warrants consideration. In traditional ginseng preparations, F1, Rg5, Rk1, Rh1, and Rg2 coexist and may exhibit entourage effects through complementary mechanisms: F1’s immunostimulation could prime the immune system while Rg5/Rk1/Rh1/Rg2 prevent excessive inflammation. This multi-component interaction mirrors the traditional use of whole ginseng extracts and suggests that optimal therapeutic outcomes might require carefully designed combinations rather than single compounds. Future studies should investigate these entourage effects using systematic combination matrices and isobologram analyses to identify optimal ratios and understand the mechanistic basis of multi-ginsenoside interactions.

**Figure 4:**
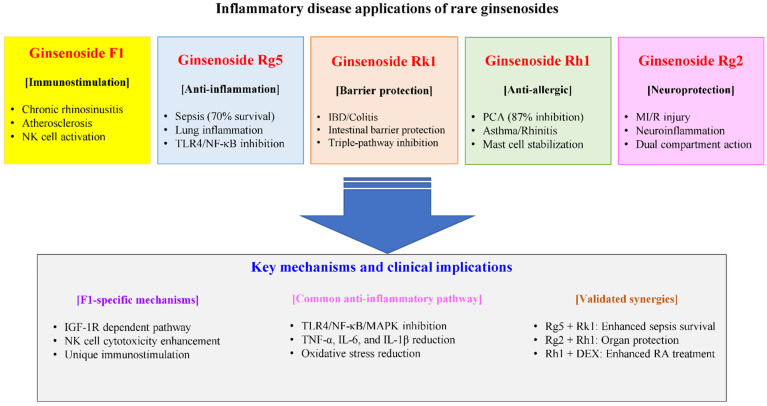
Inflammatory disease applications and immunomodulatory mechanisms of rare ginsenosides. F1 demonstrates unique immunostimulatory effects through NK cell activation, contrasting with the anti-inflammatory properties of Rg5, Rk1, Rh1, and Rg2. The bottom panel presents key mechanisms in three categories: F1-specific immunostimulatory pathways, common anti-inflammatory pathways, and validated synergistic effects. IBD, inflammatory bowel disease; MI/R, myocardial ischemia/reperfusion; PCA, passive cutaneous anaphylaxis; NK, natural killer; IGF-1R, insulin-like growth factor-1 receptor; TLR4, toll-like receptor 4; NF-κB, nuclear factor-kappa B; MAPK, mitogen-activated protein kinase; TNF-α, tumor necrosis factor-alpha; IL, interleukin; DEX, dexamethasone; RA, rheumatoid arthritis.

To provide a comprehensive overview of the inflammatory disease applications of these rare ginsenosides, we have summarized their therapeutic targets and mechanisms in Figure 4. The analysis reveals F1’s unique immunostimulatory role contrasting with the anti-inflammatory effects of Rg5, Rk1, Rh1, and Rg2. These distinct but complementary mechanisms suggest potential for both individual and combination therapeutic strategies in inflammatory diseases.

## 7. From Bench to Bedside: Translational Challenges and Opportunities

### 7.1. Current Translational Status

The translation of ginsenosides from bench to bedside faces significant challenges despite extensive preclinical evidence. Understanding the current status requires examining both successful natural product paradigms and the specific obstacles hindering ginsenoside clinical development.

Established natural products provide instructive benchmarks for therapeutic translation timelines. The nanoparticle albumin-bound paclitaxel formulation (Abraxane) received Food and Drug Administration (FDA) approval in 2005, achieving significantly higher tumor response rates (33% vs. 19%) and longer time to tumor progression (23.0 vs. 16.9 weeks) compared to standard paclitaxel in metastatic breast cancer patients [210]. Since the approval of Doxil in 1995, several nanodrugs have received FDA approval for clinical use, including small interfering RNA-delivered Onpattro and coronavirus disease 2019 (COVID-19) vaccines Comirnaty and Spikevax [210].

Clinical applications across diverse therapeutic areas demonstrate broad therapeutic potential. Polyethylene glycol-conjugated (PEGylated) nanoparticle albumin-bound steroidal ginsenoside derivatives alleviated severe acute respiratory syndrome coronavirus 2 (SARS-CoV-2)-mediated hyper-inflammatory responses in intensive care unit (ICU) patients, effectively reducing histone H4 and neutrophil extracellular traps formation (NETosis)-related factors in plasma [211]. A 4-week clinical pilot study with 68 postmenopausal women with hypercholesterolemia demonstrated that Korean red ginseng significantly reduced serum ceramide levels, particularly C16 ceramide (d18:1/16:0: −6.4 ± 6.3 pmol/mL vs. 14.6 ± 6.8 pmol/mL, *p* = 0.040) [212]. The “three syndromes and six Chinese patent medicines” randomized, double-blind, placebo-controlled, multicenter clinical trial showed that ginsenoside-containing formulations significantly improved cardiopulmonary function, sleep disorders, and digestive function [213].

The fundamental translational challenge stems from complex pharmacokinetic properties that create unpredictable therapeutic responses. Human pharmacokinetic studies following repeated oral administration of red ginseng extract revealed selective absorption patterns: among 13 ginsenosides tested, only nine (Rb1, Rb2, Rc, Rd, Rg3, Compound K, Rh2, PPD, and PPT) appeared in plasma samples, while major ginsenosides like Re and Rh1 remained undetected despite their high content in red ginseng extract [39]. Metabolites Compound K, Rh2, PPD, and PPT appeared although not present in the original extract, indicating extensive intestinal metabolism via gut microbiota. Clinical studies in metabolic disorders confirmed this biotransformation variability, as ginsenosides Rb1, Rb2, and Re remained undetected in plasma after treatment with ginseng root extract or ginsenoside Re in overweight and obese participants with impaired glucose tolerance [214].

To address these pharmacokinetic limitations, researchers have developed innovative delivery systems. Ginsenoside Rg3-based systems for pH-responsive drug delivery show promise in reducing carrier toxicity while maintaining therapeutic efficacy [215]. These formulation advances represent critical steps toward overcoming the bioavailability barriers that have historically limited clinical translation.

Safety profiles from available clinical studies suggest favorable tolerability within tested parameters. A subchronic toxicological study of the ginsenoside derivative 25-methoxy-protopanaxadiol (25-OCH3-PPD) in beagle dogs found no adverse effects at doses up to 240 mg/kg per day, with no genotoxic properties observed [216]. A randomized, double-blind, crossover study with 70 rheumatoid arthritis patients showed no significant association between Korean Red Ginseng treatment and disease flare rate (flare rate 3.7% in each group) [217]. While these studies provide valuable safety data, the structural diversity within the ginsenoside class necessitates individual safety assessment for each compound and therapeutic application.

Current clinical development strategies emphasize combination therapies and specialized formulations rather than single molecular entities, as evidenced by the clinical success of Xuesaitong for stroke treatment and ginsenoside-containing medicines for COVID-19 recovery [213]. This approach leverages the multi-target properties of ginsenosides while addressing the challenges of standardization and bioavailability.

### 7.2. Pharmacokinetic Challenges and Solutions

The clinical translation of rare ginsenosides is fundamentally limited by poor oral bioavailability, a challenge that affects all five compounds but is being addressed through innovative formulation strategies.

Ginsenoside F1 exemplifies the bioavailability challenge and its potential solutions. F1 demonstrates only 26.0% permeability across intestinal epithelial barriers, which nanostructured lipid carrier formulations improve to 39.2% with 90% encapsulation efficiency [85]. While direct pharmacokinetic data for F1 in its isolated form are limited, studies of its precursor Rg1 provide insights—when Rg1 (25 mg/kg) is orally administered in rats, 40.11% is excreted in feces with F1 detected as one of the metabolites alongside Rh1 (22.19%) and protopanaxatriol (22.88%), while urinary excretion remains minimal (0.04%) [83]. Similarly, ginsenoside Rg5 shows minimal cellular uptake in its free form, with metabolic studies in zebrafish revealing extensive biotransformation into seven metabolites through desugarization, glucuronidation, and sulfation pathways [218].

Biotechnological approaches offer scalable solutions for production challenges. Computer-guided enzyme engineering achieved a 13.88-fold increase in F1 biosynthesis efficiency through β-glucosidase optimization, with food-grade *Corynebacterium glutamicum* expression systems maintaining 75.4% activity compared to laboratory strains [192]. These advances enable pharmaceutical-grade manufacturing while meeting regulatory requirements.

The incorporation of ginsenosides into liposomal membranes creates multifunctional delivery platforms with particle sizes of 100–200 nm and enhanced stability profiles [219]. These formulation strategies address both bioavailability limitations and production scalability for F1 and Rg5.

Ginsenoside Rk1 faces similar bioavailability challenges with oral bioavailability of only 2.87–4.23% in rats (T_1_/_2_: 3.09-3.40 h, Tmax: 4.29–4.57 h) [118] and extensive metabolism into four metabolites in zebrafish [218]. To address this limitation, Rk1-loaded liposomes have been developed, achieving 97.24% encapsulation efficiency. These modified liposomes utilize exposed Rk1 glycosides to reduce plasma protein adhesion and bind to GLUT1 protein highly expressed in tumor cells, resulting in enhanced tumor accumulation and over 50% reduction in tumor volume compared to conventional formulations [117].

For ginsenoside Rh1, pharmacokinetic studies revealed extremely poor bioavailability—only 1.01% with rapid elimination (T_1_/_2_β: 0.41 ± 0.05 h) due to extensive presystemic metabolism [220]. SMEDDS formulations with CYP450 and P-gp inhibitory excipients have demonstrated significant improvement, achieving up to 33.25% bioavailability [139].

Ginsenoside Rg2 has been successfully formulated in lipid nanoparticles that achieve 81.9% mRNA encapsulation efficiency through hydrogen bond formation between Rg2’s hydroxyl groups and mRNA’s polar functional groups. Metabolically, Rg2 undergoes CYP450-mediated oxidation at the C17-side-chain, producing metabolites (M1, M3, M4) with superior SIRT1 activation compared to the parent compound [161], and in humans, is detected only as metabolites in plasma following oral red ginseng administration [39]. These formulations demonstrate the potential for Rg2 not only as a therapeutic agent but also as a functional excipient in advanced drug delivery systems [162]. The pharmacokinetic challenges and successful formulation strategies for improving bioavailability are summarized in Table 4. However, it should be noted that comprehensive human pharmacokinetic parameters remain limited for these compounds, with most data derived from animal models, highlighting the critical need for clinical pharmacokinetic studies.

### 7.3. Critical Assessment of Delivery Systems: Benefits, Limitations, and Enhancement Strategies

The clinical translation of rare ginsenosides faces a fundamental challenge: despite remarkable pharmacological activities, their oral bioavailability remains below 5% due to inherent physicochemical limitations. Ginsenosides exhibit poor membrane permeability due to their high molecular weight, exemplified by ginsenoside Rb1 (1109.46 Da) achieving only 4.35% bioavailability in rats [221]. Additionally, P-glycoprotein efflux systems actively expel ginsenosides from intestinal cells, while gastric acid and intestinal microflora degrade these compounds through hydrolysis and deglycosylation at C-3 and C-20 positions [221]. These obstacles necessitate innovative delivery strategies to unlock their therapeutic potential.

Current nanoformulation approaches have achieved varying degrees of success in overcoming these limitations. Liposomal systems demonstrate the most versatile improvements, with ginsenoside Rk1-loaded liposomes achieving 97.24% encapsulation efficiency while utilizing exposed Rk1 glycosides to reduce plasma protein adhesion and bind to GLUT1 proteins overexpressed on tumor cells [222]. Remarkably, ginsenoside Rg3 can replace cholesterol as a membrane stabilizer due to its similar steroid structure, eliminating cholesterol-related cardiovascular risks while maintaining liposomal integrity [192]. However, liposomes face storage instability issues and scale-up manufacturing challenges that limit their clinical translation.

Self-microemulsifying drug delivery systems (SMEDDS) have demonstrated superior bioavailability enhancement among all formulation strategies. Ginsenoside Rh1 SMEDDS incorporating both CYP450 and P-glycoprotein inhibitory excipients achieved 33.25% oral bioavailability compared to 12.92% for free drug, representing a 2.6-fold improvement [221]. These systems spontaneously form microemulsions in the gastrointestinal tract, dramatically increasing the interfacial surface area for absorption [223]. The limitation lies in high surfactant concentrations (up to 33.33% Tween 20) that may cause gastrointestinal irritation [224].

Nanostructured lipid carriers (NLC) offer advantages in drug stability and controlled release. F1-loaded NLC achieved 90% encapsulation efficiency with particle sizes averaging 98.9 nm, increasing Caco-2 cell permeability from 26.0% to 39.2% [221]. The aerosol solvent extraction system (ASES) technology transforms ginsenosides from crystalline to amorphous state, with polymer excipients preventing recrystallization through hydrogen-bonding interactions, thereby improving dissolution rates [225]. Nevertheless, maintaining long-term stability of amorphous formulations remains challenging.

Polymeric nanoparticles enable targeted delivery through surface functionalization. Cyclodextrin inclusion complexes demonstrated significant bioavailability enhancement, with γ-cyclodextrin complexation increasing ginsenoside Re solubility by 1.8-fold and achieving 171% relative bioavailability compared to free drug [226]. Similarly, mucoadhesive enteric-coated microparticles showed that protopanaxadiol-type saponins achieved higher encapsulation efficiency than protopanaxatriol-type due to their greater lipid solubility [227]. The complexity of achieving consistent batch-to-batch reproducibility limits their industrial scalability

These delivery systems enhance bioavailability through multiple mechanisms. P-glycoprotein inhibition by excipients such as TPGS and Pluronic polymers prevents drug efflux, as demonstrated by HM30181 increasing paclitaxel bioavailability from 3.4% to 41.3% when combined with P-gp substrates [228]. Lipid-based formulations promote lymphatic uptake, bypassing first-pass hepatic metabolism [228]. Nanovesicles facilitate transcellular transport through clathrin- and caveolin-mediated endocytosis pathways, with their structural flexibility and intestinal epithelial affinity markedly augmenting oral bioavailability [229].

Recent innovations point toward more sophisticated enhancement strategies. Ginsenoside compound K-based multifunctional liposomes utilize CK simultaneously as therapeutic agent and membrane stabilizer, eliminating the need for cholesterol while maintaining structural integrity [230]. Co-delivery approaches demonstrate synergistic benefits, with ginsenoside Rg3 co-administered with paclitaxel achieving 3.4-fold higher bioavailability through competitive P-gp binding [231]. pH-responsive systems exploiting the acidic tumor microenvironment for triggered drug release represent promising future directions [232].

The path forward requires addressing key challenges: standardization of evaluation methods for ginsenoside formulations, development of scalable manufacturing processes maintaining quality while reducing costs, and personalized dosing strategies accounting for inter-individual variability in gut microbiota-mediated metabolism. These integrated approaches will be essential for translating the therapeutic potential of rare ginsenosides into clinical reality.

### 7.4. Pharmaceutical Applications and Safety Profiles

The therapeutic landscape for the conditions discussed in this review is dominated by synthetic pharmaceuticals with well-documented side effect profiles. In Alzheimer’s disease treatment, acetylcholinesterase inhibitors show significant tolerability issues. Donepezil demonstrates discontinuation rates ranging from 20.9% in prospective studies to over 40% in retrospective analyses, with adverse events including nausea, vomiting, diarrhea, bradycardia, and insomnia [233]. Cardiac side effects including QT-prolongation and symptomatic bradycardia have been reported [234]. Memantine causes agitation, somnolence, and confusion with discontinuation rates around 10% [235]. In cancer treatment, chemotherapy toxicities vary by regimen. For non-small cell lung cancer, paclitaxel-carboplatin results in grade 3/4 neutropenia in 50% of patients, with 28.6% experiencing severe peripheral neuropathy [236]. In urothelial carcinoma, paclitaxel-doxorubicin combinations show neutropenia (58.3%) and required dose reductions in 16.7% of patients [237]. Metformin for diabetes, while generally well-tolerated, carries the risk of lactic acidosis with an incidence of 1–5 cases per 100,000 patient-years and mortality rates of 25–50% when it occurs [238]. A case report documented severe concurrent lactic acidosis and ketoacidosis with lactate levels reaching 17.5 mmol/L [239]. For hepatocellular carcinoma, standard transcatheter arterial chemoembolization (TACE) therapy shows significant adverse events including fever, abdominal pain, and vomiting in 44.7% of patients [240].

In contrast, clinical studies of ginsenosides demonstrate favorable safety profiles. A randomized trial of Korean Red Ginseng in 180 participants showed no significant adverse events at doses up to 3.6 g/day for 4 weeks, with only mild gastrointestinal symptoms reported [241]. Intravenous ginsenoside Rd showed good tolerability with transient, self-resolving white blood cell count decreases [242]. Large-scale studies support this safety profile. A multicenter trial of *Panax notoginseng* saponins in 3,072 ischemic stroke patients reported serious adverse events in only 1.0% versus 1.1% in controls [243]. *Panax ginseng* extract in hepatic dysfunction showed adverse events similar to placebo [244]. Notably, ginsenoside Rg3 combined with TACE actually reduced treatment-related adverse events compared to TACE alone, with lower rates of vomiting (12.5% vs. 44.7%) and improved tolerability [240]. Phase I trials of compound K demonstrated no dose-limiting toxicities up to 800 mg with primarily mild gastrointestinal symptoms [245]. However, comprehensive long-term safety data from Phase III trials remain limited for isolated rare ginsenosides compared to established pharmaceuticals, necessitating continued vigilance and further investigation.

The safety and toxicity profiles of rare ginsenosides have been evaluated individually through preclinical and clinical studies. Ginsenoside F1 has demonstrated safety in neuroprotective applications, though specific toxicity data remain limited; available studies focus primarily on its metabolic effects without reporting adverse events [24]. Ginsenoside Rg5 has been systematically reviewed, showing anticancer effects at concentrations of 25-100 μM without cytotoxicity to normal cells, though comprehensive toxicity studies are still needed [95,107]. Ginsenoside Rk1, often studied in combination with Rg5, demonstrated hepatoprotective effects at 10 mg/kg in acetaminophen-induced liver injury models without observable toxicity [107]. Ginsenoside Rh1 showed no adverse effects in anti-inflammatory applications and enhanced dexamethasone’s therapeutic effects without increasing its toxicity [130]. Ginsenoside Rg2 exhibited the most comprehensive safety profile, with no acute oral toxicity at single doses up to 10 g/kg and no subchronic toxicity at 5 g/kg/day for 28 days; it also demonstrated cardioprotective effects without proarrhythmic risks [32].

Clinical trials have validated the safety of ginsenoside-containing preparations. Xuesaitong injection containing *Panax notoginseng* saponins showed adverse drug reaction rates of only 4.14‰ in 30,884 patients, with most reactions being mild and reversible [246]. Red ginseng extract containing multiple rare ginsenosides was safely administered at 3 g/day to acute myocardial infarction patients for 8 months without serious adverse events [247]. Ginseng berry extract and sprout extract demonstrated safety in 12-week trials with no significant differences in adverse events compared to placebo [248,249].

Standardization and quality control are achieved through validated analytical methods. High-performance liquid chromatography coupled with mass spectrometry (HPLC-MS) enables simultaneous quantification of over 23 ginsenosides with detection limits in the nanogram range [250]. The International Organization for Standardization has registered manufacturing processes ensuring ginsenoside content variations within ± 10% [251]. Quality markers include the Rg1/Rb1 ratio for species identification and total ginsenoside content ranging from 2–20% depending on processing methods [250]. Despite these advances, complete toxicological profiles including genotoxicity, reproductive toxicity, and carcinogenicity studies for individual rare ginsenosides F1, Rg5, and Rk1 remain to be fully established.

### 7.5. Future Opportunities and Perspectives

Despite significant advances in understanding the immunomodulatory mechanisms of emerging rare ginsenosides, substantial opportunities remain for optimizing their therapeutic translation. The distinct mechanisms identified in Section 3, Section 4, Section 5 and Section 6 provide a roadmap for targeted development strategies. Building on the pharmacokinetic challenges discussed in Section 7.2, advanced formulation technologies offer the most immediate path to clinical translation for F1, Rg5, Rk1, Rh1, and Rg2.

Recent developments in nanoformulation technologies offer promising solutions for the bioavailability challenges inherent to rare ginsenosides. Ginsenoside F1 encapsulated in nanostructured lipid carriers achieved 39.2% Caco-2 cell permeability compared to 26.0% for free F1, with 90% encapsulation efficiency [85]. Production efficiency has similarly advanced through computer-guided enzyme engineering, achieving a 13.88-fold increase in F1 biosynthesis efficiency through β-glucosidase optimization [53]. For other compounds, liposomal formulations achieved 97.24% encapsulation efficiency with enhanced tumor targeting for Rk1 [117], while SMEDDS improved Rh1’s oral bioavailability 2.6-fold [139], and lipid nanoparticles enhanced Rg2 stability [162]. These successes across all five ginsenosides demonstrate that systematic application of advanced formulation strategies could substantially improve their therapeutic potential.

Sustainable large-scale production remains essential for clinical feasibility. Enzymatic bioconversion of ginseng powder containing F1 resulted in 1.5- to 2-fold higher frequency of NK1.1high cells in treated mice [63]. For Rk1 production, ultrasonication-processed red ginseng extracts optimized at 100 °C for 12 h achieved the highest concentrations at 0.175% [207]. The use of GRAS host strains such as *Corynebacterium glutamicum* for expressing ginsenoside-converting enzymes enabled food-grade production of rare ginsenosides, with enzyme activities reaching 75.4% compared to *Escherichia coli* expression systems [52]. These biotechnological advances, combined with high-speed counter-current chromatography for purification [123] and controlled steaming processes for Rg5 [47], establish viable pathways for pharmaceutical-scale manufacturing.

The availability of standardized production enables exploration of synergistic combinations, leveraging the complementary mechanisms identified in Section 4. The Rg5/Rk1 combination achieved 70% survival rate in septic models compared to 20% in controls at 20 mg/kg [66]. Similarly, Rg2 and Rh1 demonstrated empirical synergy through co-administration, attenuating LPS-induced inflammation through selective inhibition of p38 MAPK and STAT1 activation [42]. Notably, Rh1 potentiated dexamethasone’s anti-inflammatory effects while reversing glucocorticoid resistance [130]. These empirically validated synergistic interactions suggest that rational combination design based on complementary mechanisms could maximize therapeutic outcomes while potentially reducing individual compound doses.

Emerging therapeutic applications extend beyond traditional inflammatory diseases. Rg5’s specific activation of IGF-1 receptor at EC50 ~90 nM opens possibilities for angiogenesis and vascular disorders [65]. Its ability to ameliorate insulin resistance by modulating gut microbiota suggests metabolic disease applications [103]. F1’s unique NK cell enhancement positions it for cancer immunotherapy, particularly when combined with checkpoint inhibitors [25]. Rh1’s specialized anti-allergic properties offer precision medicine approaches for allergic diseases [138], while Rg2’s dual neuroimmune modulation provides opportunities in neuroinflammatory conditions [42]. The Rg5/Rk1 combination’s effects on GABAergic/serotoninergic signaling and osteoblast proliferation suggest applications in sleep disorders and osteoporosis [99].

Clinical translation pathways benefit from established safety profiles and existing regulatory frameworks. Subchronic toxicological studies found no adverse effects at doses up to 240 mg/kg per day [216], while clinical trials in rheumatoid arthritis patients showed no significant disease flare associations [217]. The GRAS status of ginseng facilitates regulatory development [44], with standardized red ginseng preparations already achieving 1.0% Rk1 content [120].

The significant inter-individual variability in ginsenoside metabolism, driven by differences in gut microbiota composition, necessitates personalized therapeutic approaches. Since intestinal bacteria-mediated biotransformation determines the actual bioactive metabolites produced, patients with different microbiome profiles may experience vastly different therapeutic outcomes from the same ginsenoside. This underscores the need for either microbiome-based diagnostic tools to predict individual metabolic patterns or the development of pre-converted formulations containing the active metabolites directly, bypassing the need for microbial transformation.

Despite these advances, several critical aspects remain unclear and require further investigation. The precise molecular mechanisms underlying the synergistic effects between rare ginsenosides, particularly the Rg5/Rk1 combination’s 70% survival rate in septic models, have not been fully elucidated at the systems biology level. The role of gut microbiota in determining individual therapeutic responses remains poorly understood, with no established biomarkers to predict which patients will be responders versus non-responders. Long-term safety profiles of nanoformulated ginsenosides, especially regarding potential accumulation in specific organs and immunogenicity after repeated administration, require systematic evaluation. The optimal dosing regimens for combination therapies, including timing and sequence of administration when combined with checkpoint inhibitors or biologics, have not been established. Furthermore, the economic feasibility of industrial-scale production while maintaining quality standards remains unresolved, particularly for maintaining batch-to-batch consistency of complex nanoformulations. These knowledge gaps highlight the need for integrated approaches combining systems pharmacology, microbiome analysis, and advanced manufacturing technologies to fully realize the therapeutic potential of rare ginsenosides.

The unique immunomodulatory profiles of these rare ginsenosides present compelling opportunities for combination with existing immunotherapies. F1’s NK cell-enhancing properties could synergize with immune checkpoint inhibitors such as PD-1/PD-L1 antibodies, potentially overcoming resistance mechanisms in patients with low NK cell activity. The combination could address both adaptive immunity (checkpoint inhibitors) and innate immunity (F1-mediated NK activation), providing a more comprehensive anti-tumor response. Similarly, the potent anti-inflammatory effects of Rg5/Rk1 could complement anti-TNF biologics in treating inflammatory diseases, potentially allowing dose reduction of expensive biologics while maintaining therapeutic efficacy. The multi-pathway inhibition by Rk1 could address inflammatory mechanisms not targeted by TNF blockade alone. Rh1’s anti-allergic properties might enhance the efficacy of antihistamines or leukotriene inhibitors in severe allergic conditions, while Rg2’s neuroprotective effects could be combined with existing treatments for neurodegenerative diseases. These combination strategies warrant systematic investigation through preclinical studies to establish optimal dosing regimens, timing of administration, and safety profiles before clinical translation.

Moving forward, several strategic priorities emerge. First, developing patient biomarkers for personalized ginsenoside selection could optimize therapeutic outcomes. Second, establishing cost-effective production methods would ensure global accessibility. Third, conducting head-to-head comparative studies would enable precise therapeutic positioning. Finally, exploring combination therapies with conventional drugs could leverage the unique mechanisms of each ginsenoside. Collectively, these advances position F1, Rg5, Rk1, Rh1, and Rg2 for accelerated clinical translation, with F1’s unique immunostimulatory properties offering particular promise for combination immunotherapy while the other four ginsenosides provide targeted anti-inflammatory approaches for precision medicine applications.

## 8. Conclusions

This comprehensive review has examined the emergence of five rare ginsenosides—F1, Rg5, Rk1, Rh1, and Rg2—as promising immunomodulatory agents distinct from their major ginsenoside precursors. These compounds, produced through deglycosylation, heating, and steaming processes of *Panax ginseng*, represent a new generation of bioactive molecules with enhanced therapeutic potential. The transformation from major to rare ginsenosides not only improves bioavailability and membrane permeability but also fundamentally alters their immunological activities, creating opportunities for targeted therapeutic interventions. Mechanistic studies revealed a fundamental dichotomy: ginsenoside F1 uniquely functions as an immunostimulant through MAPK/NF-κB activation, enhancing NK cell cytotoxicity and macrophage phagocytosis, while Rg5, Rk1, Rh1, and Rg2 exhibit anti-inflammatory properties through distinct mechanisms. SAR analysis demonstrated that sugar moiety position and number critically determine these divergent effects. F1’s C-20 glucose confers immunostimulation, while different sugar arrangements in other compounds direct anti-inflammatory functions. These distinct mechanisms—from Rg5’s TLR4 antagonism to Rk1’s multi-pathway modulation, Rh1’s selective kinase inhibition, and Rg2’s dual neuroimmune regulation—provide a molecular basis for compound-specific therapeutic applications. Clinical evidence supports therapeutic efficacy across diverse conditions, with favorable safety profiles from preclinical and clinical studies. Importantly, breakthrough advances in delivery systems—including NLC achieving 39.2% permeability for F1, SMEDDS providing 2.6-fold bioavailability enhancement for Rh1, and liposomal formulations with 97% encapsulation for Rk1—have transformed these compounds from laboratory curiosities to clinically viable candidates. These delivery innovations represent the critical enabling technology that bridges the gap between promising preclinical data and practical therapeutic applications. The multi-pathway modulation capabilities demonstrated by these compounds, particularly Rk1’s ability to simultaneously target NF-κB, MAPK, and STAT signaling pathways, represent a significant pharmacological advantage over conventional single-target synthetic drugs. This aligns with modern polypharmacology approaches that recognize complex diseases require multi-target intervention to prevent compensatory mechanisms and achieve comprehensive therapeutic effects. Future strategies should leverage F1’s unique immunostimulatory properties for combination cancer immunotherapy while utilizing the complementary anti-inflammatory mechanisms of Rg5, Rk1, Rh1, and Rg2 for precision medicine approaches in inflammatory and metabolic diseases. The integration of advanced formulation technologies with optimized production methods, coupled with established safety profiles and regulatory frameworks for ginseng products, creates a clear pathway for clinical translation. However, it must be acknowledged that despite compelling preclinical evidence across multiple disease models, robust human clinical trial data specifically for F1, Rg5, Rk1, Rh1, and Rg2 remains largely absent. Furthermore, while available safety data is encouraging, comprehensive toxicological profiles for each ginsenoside, especially regarding chronic administration and potential drug interactions, require systematic evaluation. The majority of evidence derives from in vitro studies and animal models, with human data limited to pharmacokinetic studies and safety assessments rather than efficacy trials. This critical gap between preclinical promise and clinical validation represents the most significant hurdle for therapeutic development and underscores the urgent need for well-designed phase I/II clinical trials to establish safety, optimal dosing, and preliminary efficacy in human subjects. Furthermore, several bottlenecks must be addressed for successful translation to clinical therapeutics. Production scalability remains challenging despite advances in enzymatic conversion and biotechnological approaches described in Section 2.1.3. The significant inter-individual variability in gut microbiota-mediated biotransformation complicates standardization and dosing strategies (Section 7.3). Bioavailability challenges require continued optimization of advanced delivery systems including NLC, SMEDDS, and liposomal formulations (Section 7.2). Additionally, intellectual property protection for naturally occurring compounds and unclear regulatory pathways for botanical-derived drugs present commercial development challenges. These five rare ginsenosides thus represent a new frontier in immunomodulatory therapeutics, offering targeted solutions for immune-related disorders.

## Figures and Tables

**Table 1:** Structural characteristics and immunomodulatory properties of five rare ginsenosides.

Ginsenoside	Type	Key Immunomodulatory Effects	Main Mechanisms	Ref.
F1	PPT	NK cell activation Enhanced cytotoxicity	IGF-1-dependent pathway	[25,27,63]
Rg5	PPD	Anti-inflammatory Reduced LPS-induced cytokines	TLR4/NF-κB inhibition IGF-1R activation (EC50: 90 nM)	[64,65]
Rk1	PPD	Anti-inflammatory Suppressed HMGB1 responses	SIRT1-mediated HMGB1 deacetylation NF-κB/ERK1/2 inhibition	[66]
Rh1	PPT	Anti-inflammatory Antioxidant Reduced microglial activation	ROS scavenging Suppression of microglial activation (OX-42) p38 MAPK/STAT1 inhibition (with Rg2)	[41,42]
Rg2	PPT	Anti-inflammatory Reduced LPS-induced cytokines Inhibited inflammatory mediators	p38 MAPK/STAT1 inhibition NF-κB pathway suppression PKCδ translocation blockade	[42,67]

PPT, protopanaxatriol; PPD, protopanaxadiol; NK, natural killer; EC50, half maximal effective concentration; IGF-1, insulin-like growth factor-1; IGF-1R, IGF-1 receptor; LPS, lipopolysaccharide; TLR4, Toll-like receptor 4; NF-κB, nuclear factor-κB; MAPK, mitogen-activated protein kinase; HMGB1, high-mobility group box 1; SIRT1, sirtuin 1; ERK, extracellular signal-regulated kinase; ROS, reactive oxygen species.

**Table 2:** Immunomodulatory effects and clinical applications of five rare ginsenosides. The table summarizes the primary immunological effects, key molecular mechanisms, target diseases, and supporting references for ginsenosides F1, Rg5, Rk1, Rh1, and Rg2. F1 uniquely demonstrates immunostimulatory properties through NK cell activation, contrasting with the anti-inflammatory effects of the other four compounds. NK, natural killer; TLR4, Toll-like receptor 4; NF-κB, nuclear factor-kappa B; NLRP3, NOD-like receptor protein 3; MAPK, mitogen-activated protein kinase; STAT1, signal transducer and activator of transcription 1; IBD, inflammatory bowel disease; AD, Alzheimer’s disease.

Ginsenoside	Primary Effect	Key Mechanisms	Target Diseases	Ref.
F1	Immunostimulation	NK cell activation Macrophage enhancement	Cancer Immunodeficiency	[25,63]
Rg5	Anti-inflammation	TLR4/NF-κB inhibition	Sepsis Metabolic disorders	[66,103]
Rk1	Multi-pathway inhibition	NLRP3, MAPK modulation	Arthritis IBD	[66,117]
Rh1	Anti-allergic	p38 MAPK, STAT1 inhibition	Asthma Allergic diseases	[42,130]
Rg2	Neuroimmune regulation	Dual compartment modulation	Neuroinflammation AD	[42,162]

**Table 3:** Summary of in vivo efficacy data for rare ginsenosides. i.p., intraperitoneal; i.v., intravenous; p.o., per oral; CLP, cecal ligation and puncture; CIA, collagen-induced arthritis; MCAO, middle cerebral artery occlusion; MVD, microvessel density; DEX, dexamethasone.

Model	Ginsenoside	Dose	Route	Duration	Key Outcome	Ref.
**Cancer models**						
CT26 colon cancer (mice)	F1	20 mg/kg	i.p.	14 days	67% tumor growth inhibition	[25]
RMA-s lymphoma (mice)	F1	25–50 mg/kg	i.p.	3 days pretreatment	70% rejection vs. 20% control at 6 h	[25]
B16F10 melanoma (mice)	F1	50 mg/kg	i.p.	3 days pre + 3×/week	Reduced lung metastases	[25]
**Sepsis models**						
CLP-induced sepsis (mice)	Rg5/Rk1	0.061 mg/kg	i.v.	12 h & 50 h post-CLP	70% survival vs. 20% control	[66]
HMGB1-induced sepsis (mice)	Rg5/Rk1	0.031–0.061 mg/kg	i.v.	Single	Reduced HMGB1, TNF-α, IL-6	[66]
**Inflammatory models**						
LPS-induced inflammation (mice)	Rg2/Rh1	Not specified	Not specified	Not specified	Synergistic reduction in liver/kidney damage	[42]
CIA (mice)	Rh1 + DEX	10 mg/kg (Rh1) + 1 mg/kg (DEX)	i.p.	10 days	Enhanced anti-inflammatory effects vs. DEX alone	[130]
**Neurological models**						
MCAO stroke (rats)	F1	50 mg/kg	p.o.	14 days	Increased MVD, improved cerebral perfusion	[26]
Sleep deprivation (mice)	Rg5/Rk1	30–60 mg/kg	p.o.	7 days	Increased sleep duration, reduced latency	[99]

**Table 4:** Pharmacokinetic parameters and bioavailability enhancement strategies for five rare ginsenosides. NLC, nanostructured lipid carrier; SMEDDS, self-microemulsifying drug delivery systems; GLUT, glucose transporter; T_1_/_2_, half-life; Tmax, time to reach maximum concentration; CYP450, cytochrome P450; P-gp, P-glycoprotein; SIRT1, sirtuin 1. Dash (–) indicates no enhancement strategy was applied in the referenced study.

Ginsenoside	Species /Model	Bioavailability /PK Parameters	Enhancement Strategy	Improved Outcome	Ref
F1	Caco-2 cells	<5% (estimated); 26.0% permeability	Nanostructured lipid carrier	39.2% permeability; 90% encapsulation	[85]
	Rat (as Rg1 metabolite)	Detected in feces (parent Rg1: 40.11%)	–	–	[83]
Rg5	In vitro	Low	Cyclodextrin complexation	1.8-fold increase	[121]
	Zebrafish	Low; 7 metabolites identified	–	–	[218]
Rk1	In vivo	<3% oral absorption	Liposome (97.24% encapsulation)	>50% tumor reduction	[117]
	Rat	2.87–4.23% (T_1_/_2_: 3.09–3.40 h)	–	–	[118]
	Zebrafish	Low; 4 metabolites	–	–	[218]
Rh1	In vitro/rat	12.92%	SMEDDS	33.25% (2.6-fold increase)	[139]
	Rat	1.01% (T_1_/_2_β: 0.41 h)	–	–	[220]
Rg2	In vitro	Low	Lipid nanoparticles	Enhanced mRNA delivery (81.9% encapsulation)	[162]
	Rat microsomes	Low; metabolites M1, M3, M4	–	–	[161]
	Human plasma	Not detected as parent	–	–	[39]

## Data Availability

Not applicable.

## Abbreviations

The following abbreviations are used in this manuscript:

25-OCH3-PPD	25-methoxy-protopanaxadiol
A20	Tumor necrosis factor-α-induced protein 3
AA	Arachidonic acid
AGS-H	Heat-processed American ginseng saponins
ALT	Alanine aminotransferase
AMPK	AMP-activated protein kinase
AP-1	Activator protein-1
ApoE	Apolipoprotein E
APPswe	Amyloid precursor protein Swedish mutation
ARE	Antioxidant response element
ASC	Apoptosis-associated speck-like protein containing a caspase recruitment domain
AST	Aspartate aminotransferase
ATP	Adenosine triphosphate
Bax	Bcl-2-associated x protein
Bcl-2	B-cell lymphoma 2
BDNF	Brain-derived neurotrophic factor
BMP-2	Bone morphogenetic protein-2
Brn-3a	Brain-specific homeobox/POU domain protein 3a
BUN	Blood urea nitrogen
C17SCV	C17 side-chain varied
cAMP	cyclic adenosine monophosphate
CD	Cluster of differentiation
c-Fos	c-Fos proto-oncogene
c-Jun	Cellular Jun proto-oncogen
c-Myc	Cellular myelocytomatosis oncogene
COL1A1	Collagen type I alpha 1
COVID-19	Coronavirus disease 2019
COX	Cyclooxygenase
cPLA2	Cytosolic phospholipase A2
CREB	cyclic adenosine monophosphate response element-binding protein
CYP3A4	Cytochrome P450 3A4
CYP450	Cytochrome P450
EC50	Half maximal effective concentration
EGCG	Epigallocatechin-3-gallate
EMT	Epithelial to mesenchymal transition
eNOS	Endothelial nitric oxide synthase
ER	Endoplasmic reticulum
Egr-1	Early growth response 1
ERK	Extracellular signal-regulated kinase
FDA	Food and Drug Administration
Foxp3	Forkhead box P3
FUNDC1	FUN14 domain containing 1
GABA	Gamma-aminobutyric acid
G-CSF	Granulocyte colony-stimulating factor
GLS1	Glutaminase 1
Glu	Glutamate
GLUT1	Glucose transporter 1
GM1	Monosialotetrahexosylganglioside 1
GRAS	Generally recognized as safe
GSH	Glutathione
GTPase	Guanosine triphosphatase
GZMB	Granzyme B
HaCaT	Human keratinocyte
HDAC2	Histone deacetylase 2
HMGB1	High mobility group box 1
HO	Heme oxygenase
hP2X7	Human P2X purinoceptor 7
HSP70	Heat shock protein 70
IBD	Inflammatory bowel disease
IC50	Half maximal inhibitory concentration
ICAM-1	Intercellular adhesion molecule-1
ICU	Intensive care unit
IDE	Insulin-degrading enzyme
IFN-γ	Interferon-gamma
Ig	Immunoglobulin
IGF-1	Insulin-like growth factor-1
IGF-1R	Insulin-like growth factor-1 receptor
IκBα	Inhibitor of nuclear factor kappa b alpha
IKKβ	Inhibitor of nuclear factor kappa b kinase subunit beta
IL	Interleukin
iNOS	Inducible nitric oxide synthase
JAK	Janus kinase
JNK	c-Jun N-terminal kinase
LOX-1	Lectin-like oxidized low-density lipoprotein receptor-1
LPS	Lipopolysaccharide
MAPK	Mitogen-activated protein kinase
MCP-1	Monocyte chemoattractant protein-1
MD2	Myeloid differentiation factor 2
MDA	Malondialdehyde
MI/R	Myocardial ischemia/reperfusion
MIP-1δ	Macrophage inflammatory protein-1 delta
MMP	Matrix metalloproteinase
MPTP	1-methyl-4-phenyl-1,2,3,6-tetrahydropyridine
MRL/lpr	Murphy Roths Large/lymphoproliferation
mTOR	Mammalian target of rapamycin
MW	Molecular weight
NADPH	Nicotinamide adenine dinucleotide phosphate
NEP	Neprilysin
NETosis	neutrophil extracellular traps formation
NeuN	Neuronal nuclei
NFATc1	Nuclear factor of activated T-cells, cytoplasmic 1
NF-κB	Nuclear factor-kappa B
NGAL	Neutrophil gelatinase-associated lipocalin
NGF	Nerve growth factor
NK	Natural killer
NKG2D	Natural killer group 2 member D
NLC	Nanostructured lipid carrier
NLRP3	Nucleotide-binding oligomerization domain-like receptor protein 3
NMDA	N-methyl-D-aspartate
NOX1	Nicotinamide adenine dinucleotide phosphate oxidase 1
Nrf2	Nuclear factor erythroid 2-related factor 2
OA	Oleanolic acid
p-ACC	Phosphoacetyl-CoA carboxylase
PAM	Positive allosteric modulator
PCA	Passive cutaneous anaphylaxis
PD-L1	Programmed death-ligand 1
PEGylated	Polyethylene glycol-conjugated
PGE	Prostaglandin E
P-gp	P-glycoprotein
PI3K/AKT	Phosphatidylinositol 3-kinase/protein kinase B
PKC	Protein kinase C
PKCδ	Protein kinase C delta
PLA2	Phospholipase A2
PLCγ	Phospholipase Cγ
PLD	Phospholipase D
PPD	Protopanaxadiol
PPT	Protopanaxatriol
PSEFS	Peanut sprout extracts cultivated with fermented sawdust medium
PSEN1dE9	presenilin 1 deletion of exon 9
RANKL	Receptor activator of nuclear factor kappa-B ligand
RC50	Half maximal response concentration
Rho	Ras homolog
RIP	Receptor-interacting protein
ROS	Reactive oxygen species
Runx2	Runt-related transcription factor 2
SAR	Structure–activity relationship
SARS-CoV-2	Severe acute respiratory syndrome coronavirus 2
SIRT1	Sirtuin 1
Smad	Smad family proteins
SMEDDS	Self-microemulsifying drug delivery systems
SOD	Superoxide dismutase
STAT	Signal transducer and activator of transcription
Syk	Spleen tyrosine kinase
TAK1	Transforming growth factor-beta-activated kinase 1
t-BHP	Tertiary-butyl hydroperoxide
TGF-β1	Transforming growth factor-β1
Th	T helper
TLR4	Toll-like receptor 4
TME	Tumor microenvironment
TNBS	Trinitrobenzenesulfonic acid
TNF-α	Tumor necrosis factor alpha
ULK1	Unc-51 like autophagy activating kinase 1
UVB	Ultraviolet B
VEGF	Vascular endothelial growth factor
ZO-1	Zonula occludens-1
α-MSH	Alpha-melanocyte stimulating hormone
α-SMA	Alpha-smooth muscle actin
γδ T cells	Epidermal gamma delta T cells
